# Extracellular vesicles as prognostic biomarkers: results of a neoadjuvant chemoimmunotherapy clinical trial in stage IIIA (N2) non-small-cell lung cancer (SAKK 16/14)

**DOI:** 10.3389/fimmu.2026.1807542

**Published:** 2026-07-01

**Authors:** Yannik da Silva, Dapi Menglin Chiang, Laura Benecke, Michael W. Pfaffl, Stefanie Hayoz, Sabrina Chiquet, Spasenija Savic Prince, Adrienne Bettini, Martin Früh, Laetitia A. Mauti, Christian Britschgi, Solange Peters, Michael Mark, Adrian F. Ochsenbein, Wolf-Dieter Janthur, Christine Waibel, Nicolas Mach, Patrizia Froesch, Martin Buess, Pierre Bohanes, Michel Gonzalez, Alfred Zippelius, Miklos Pless, Sacha I. Rothschild, Laurent Muller

**Affiliations:** 1 Department of Biomedicine, University of Basel, Basel, Switzerland; 2 Department of Otorhinolaryngology, Head and Neck Surgery, University Hospital of Basel, Basel, Switzerland; 3 Department of Animal Physiology and Immunology, Technical University of Munich (TUM) School of Life Sciences, Technical University of Munich, Freising, Germany; 4 Swiss Cancer Institute (SAKK) Competence Center, Bern, Switzerland; 5 Pathology, Institute of Medical Genetics and Pathology, University Hospital Basel, Basel, Switzerland; 6 Department of Oncology, Hôpital Fribourgeois (HFR) Fribourg – Hôpital Fribourgeois, Fribourg, Switzerland; 7 Department of Oncology/Hematology, Cantonal Hospital St. Gallen, St. Gallen, Switzerland; 8 Department of Oncology, Inselspital Bern, Bern, Switzerland; 9 Department of Oncology/Hematology, Cantonal Hospital Winterthur, Winterthur, Switzerland; 10 Department of Medical Oncology and Hematology, Comprehensive Cancer Center Zurich, University Hospital Zurich, University of Zurich, Zurich, Switzerland; 11 Department of Oncology, University Hospital Lausanne Centre hospitalier universitaire vadois (CHUV), Lausanne, Switzerland; 12 Divison of Oncology/Hematology, Cantonal Hospital Graubünden, Chur, Switzerland; 13 Department of Oncology/Hematology, Cantonal Hospital Aarau, Aarau, Switzerland; 14 Department of Oncology/Hematology, Cantonal Hospital Baden, Baden, Switzerland; 15 Department of Oncology, University Hospital Geneva, Geneva, Switzerland; 16 Oncology Institute of Southern Switzerland, Locarno, Switzerland; 17 Division of Medical Oncology, St. Claraspital, Basel, Switzerland; 18 Centre de Chimiothérapie Anti-Cancéreuse, Lausanne, Switzerland; 19 Department of Thoracic Surgery, University Hospital Lausanne Centre hospitalier universitaire vadois (CHUV), Lausanne, Switzerland; 20 Department Medical Oncology, University Hospital Basel, Basel, Switzerland

**Keywords:** biomarker, durvalumab, extracellular vesicle, neoadjuvant chemoimmunotherapy, non-small-cell lung cancer, PanCK, PD-L1

## Abstract

The introduction of immune checkpoint inhibitors has transformed cancer therapy. In non-small-cell lung cancer (NSCLC), immune-checkpoint blockade targeting programmed cell death 1 (PD-1) and its ligand PD-L1 has demonstrated substantial therapeutic efficacy and survival benefit. Recent evidence indicates that small extracellular vesicles (EVs), including exosomes, play a crucial role in modulating the tumor microenvironment and immune responses, potentially influencing immunotherapy efficacy. This study investigates longitudinal EV dynamics in patients with resectable stage IIIA (N2) NSCLC undergoing multimodal therapy, including sequential neoadjuvant chemotherapy (cisplatin and docetaxel) combined with anti-PD-L1 antibody (durvalumab) immunotherapy, followed by surgery, and adjuvant durvalumab. Serum samples from the SAKK 16/14 trial were analyzed using a galectin-based EV isolation technique. EV marker expression (PD-L1, PanEV, PanCK, EpCAM, and CD45) was assessed by flow cytometry at five predefined treatment time points. Nanoparticle tracking analysis and electron microscopy confirmed successful EV isolation. Results indicated a trend toward decreasing EV-MFI values following initial therapy. In current smokers, EV-associated markers levels correlated significantly with disease progression. Importantly, elevated post-therapeutic PanEV/PanCK double-positive EV levels were significantly associated with reduced event-free survival and overall survival (p = 0.001 and p = 0.003, respectively). This study demonstrates the feasibility of longitudinal EV biomarker assessment in NSCLC and suggests that EV-based liquid biopsies may provide complementary prognostic information in the context of cancer therapy. Such approaches may be particularly informative in heterogeneous cancer stages, supporting future efforts toward personalized treatment strategies.

## Introduction

Lung cancer remains the leading cause of cancer-related mortality, accounting for approximately 18% of all cancer deaths in 2020. Notably, over 80% of lung cancer-related deaths are attributed to non-small cell lung cancer (NSCLC) (1). In recent years, therapeutic strategies have evolved significantly, with an increasing focus on immunotherapies and molecularly targeted treatments, either as standalone options or in combination with conventional approaches such as radiotherapy, chemotherapy, and surgery (2, 3). Despite these advances, long-term survival remains poor, emphasizing the urgent need for better prognostic markers to identify responders and optimize treatment strategies.

Resectable stage IIIA (N2) NSCLC exemplifies these challenges. This entity represents a highly heterogenous subgroup in which individualized treatment strategies must be determined in a multidisciplinary framework. The optimal standard of care remains under international debate and currently ranges from neoadjuvant chemoimmunotherapy followed by surgical resection with adjuvant chemotherapy to definitive radiotherapy alone (4). With the expanding use of neoadjuvant systemic therapies, tumors initially classified as unresectable may be downstaged, thereby enabling curative-intent surgery and potentially improving prognosis (5). Although multimodal imaging techniques, including PET/CT, CT, MRI, and invasive staging procedures such as mediastinoscopy or endobronchial ultrasound–guided fine-needle aspiration, are widely accepted for assessing resectability and disease stage, significant limitations persist. To date, a universally validated biomarker capable of predicting the response to a given therapeutic modality and reliably evaluating prognostic treatment outcomes has not been established. Micrometastatic disease may be missed, inflammatory lesions can mimic malignancy, and false-negative cytological results may obscure residual tumor burden, highlighting the need for additional diagnostic and prognostic biomarkers to enhance staging accuracy and personalized treatment stratification.

The field of diagnostics in cancer is undergoing continuous advancements. Beyond traditional tissue biopsies, liquid biopsy techniques – including the analysis of cell-free tumor DNA (ctDNA), microRNA, exRNA, extracellular vesicles such as exosomes, and circulating tumor cells (CTC) – are becoming increasingly important as valuable diagnostic tools (6). Small extracellular vesicles ranging from 30 to 150 nm, play a crucial role in cell-to-cell communication, immune modulation, and tumor progression (7). Since their first descriptions in 1983 by Harding and Stahl et al., as well as Pan and Johnstone et al., exosomes have been recognized for their role in the transport and presentation of the transferrin receptor (8, 9).

In oncology, EVs have been implicated in tumor progression (10), immune evasion (11), and serve as potential tools for prognosis (12, 13), staging (14, 15) and follow-up monitoring (14) in oncology. Beyond their diagnostic and prognostic potential, EVs are being investigated for therapeutic applications, including immune system modulation (16–19), vaccine development (20), and targeted drug delivery (21–23).

In the context of NSCLC, EV PD-L1 levels have been correlated with disease stage, with higher concentrations observed in patients with larger tumors, lymph node involvement, and distant metastases (24). While various studies have explored the role of EVs in oncology, no prior study has systematically examined EV levels throughout the course of chemo- and/or immunotherapy in NSCLC. However, related research has highlighted the potential of EVs as valuable circulating biomarkers in oncology. For instance, B. Han et al. have shown that microRNA in EVs collected from a tumor-draining vein after surgical resection of NSCLC could serve as a predictive marker for disease relapse (25). Similarly, Rodríguez Zorrilla et al. investigated CD63- and CAV-1-positive EV levels in patients with oral squamous cell carcinoma before and after surgery, while CAV-1-positive EV exhibited increased expression, likely due to post-surgical inflammatory responses (26). However, these studies were limited to surgical intervention and did not investigate how EV levels fluctuate in response to different treatment modalities, nor did they correlate EV markers with overall survival (OS) or event-free survival (EFS).

This study aims to evaluate the dynamics of specific EV biomarkers in liquid biopsies of NSCLC patients undergoing multimodal therapy. Serum samples were obtained from patients with resectable stage IIIA (N2) NSCLC undergoing neoadjuvant and adjuvant therapy with an anti-PD-L1 antibody (durvalumab) in addition to standard of care, neoadjuvant chemotherapy, and surgery as part of the multicenter single-arm phase II trial SAKK 16/14 (27). The biomarkers investigated include PD-L1, PanEV, PanCK, EpCAM (epithelial cell adhesion molecule), and CD45, all of which have been identified in prior research for their roles in cancer progression, therapeutic response, and prognostic significance.

Among these, PD-L1-positive EVs play a pivotal role in immune evasion and modulation of the tumor microenvironment, making them a promising target for enhancing cancer immunotherapy (24, 28, 29). EpCAM is overexpressed in epithelial malignancies, including lung cancer, where it plays a critical role in cell adhesion, proliferation, and differentiation (30, 31). PanCK is widely utilized as a marker for epithelial tumor cells (32), while CD45 serves as a pan-leukocyte marker involved in immune processes like proliferation, differentiation, and inflammation, all of which can be modulated by chemotherapy and immune response mechanisms. These processes, essential for the immune response to cancer, can be modulated by chemotherapy, potentially affecting tumor immunity (33, 34). Additionally, a PanEV-mix, consisting of CD9, CD63, and CD81 antibodies, was included as a general EV marker, as previously described by our research group (35).

By analyzing the levels and dynamics of these EV biomarkers in NSCLC patients undergoing multimodal therapy, this study seeks to determine their clinical relevance in treatment monitoring and outcome prediction. Especially in the context of immunotherapy, establishing reliable and appropriate tools for therapy monitoring remains challenging due to unconventional response patterns, including pseudo-progression, hyperprogression, and other atypical tumor dynamics (36). We hypothesize that EV marker fluctuations reflect tumor burden, immune modulation, and therapeutic efficacy (37). In particular, we anticipate that higher post-treatment PanEV/PanCK-positive EV levels will correlate with poorer survival outcomes. The inclusion of PanEV as a general EV marker allows for a more comprehensive assessment of EV dynamics during NSCLC treatment.

Our findings may contribute to the growing body of evidence supporting liquid biopsy-derived EV biomarkers in personalized oncology, paving the way for more accurate prognostic and diagnostic strategies in lung cancer management.

## Materials and methods

### Patient cohort

The multicentric, single-arm phase II trial SAKK 16/14 enrolled patients with stage IIIA NSCLC and confirmed N2 lymph node involvement who received three cycles of cisplatin and docetaxel, followed by two cycles of the anti-PD-L1 antibody durvalumab prior to tumor resection and one year of adjuvant durvalumab (27). Staging was done with PET-CT and brain MRI. N2 involvement had to be proven by mediastinoscopy or endobronchial ultrasonography with transbronchial fine-needle aspiration and was not limited to a single station. 55 patients (82%) underwent tumor resection. Under this regimen, 60% of patients achieved major pathological response (MPR), defined as less than 10% viable tumor cells in the resection specimen and 18% achieved pathological complete response (pCR), signifying an absence of viable tumor cells, respectively, as well as a 12-month EFS rate of 73%. The staging is based on the seventh edition of the TNM classification. Ethical approval (approval no. 2015-00042) was granted by the Ethics Committee of Northwestern and Central Switzerland. Written informed consent from all patients was obtained.

### Sample collection

For each patient, biospecimens were acquired at baseline (timepoint 1, TP1), post neoadjuvantchemotherapy (TP2), post-neoadjuvant durvalumab but pre-surgery (TP3) and after four cycles ofadjuvant durvalumab (TP4) and at the end of adjuvant immunotherapy (TP5) as demonstrated in **Supplementary Figure 1A**.

We received the at -80 °C banked serum samples from 20 patients enrolled in the same cohort as previously described from the SAKK 16/14 trial (27) to use as a model to test our recently published EV isolation technique (35) in a clinical trial. The samples were included based on the availability of blood samples at the relevant time points and complete clinical annotation. Patients were stratified according to clinical outcome using the same criteria as the primary endpoint of the trial. Responders were defined as patients achieving pathological complete response without relapse within 12 months after surgery, whereas non-responders were defined as patients without pCR who relapsed within 12 months. The cohort was assembled to include patients in each group to enable comparative analyses. Within these groups, samples were included based on availability and quality, without restriction for additional clinical variables. While balanced for treatment response, the cohort was otherwise kept clinically heterogeneous to reflect the broader trial population. Samples not meeting predefined quality criteria for downstream analyses were excluded.

### Galectin-based EV isolation and beads-based flow cytometry

A galectin-based isolation technique, EXÖBead, similar to the one described in ourpublication from 2022 (35), was employed in this study to isolate EVs by targeting N-linked glycoproteins, particularly galectin-3-binding protein (LGALS3BP), as outlined in the workflow for this manuscript and illustrated in **Supplementary Figure 1B**. We diluted 1 mL of serum in 0.9 mL of 0.5% EV-free bovine serum albumin (BSA) (SERVA Electrophoresis GmbH, Heidelberg, Germany) in phosphate-buffered saline (PBS) and subjected it to overnight centrifugation at 100’000 g, at 4 °C. The sample was then incubated with 50 μL of EXÖBead (1 μm, 6×10^8^ particles/mL, Biovesicle Inc., Taipei, Taiwan) for 1 hour at 37 °C. EV-EXÖBead complexes were washed twice with 1 mL of 0.5% EV-free BSA in PBS, while the magnetic beads were retained using a magnet.

### Surface marker

A 100 μL aliquot of the antibody master mix was incubated with serum EV-EXÖBead complexes for 1 hour at 37 °C. Due to limited fluorescence channels, the PanEV-antibody was used as a universal EV-marker instead of three individual antibodies. Specifically, 3 μL of PanEV-antibody was premixed with 1 μL each of PE anti-CD63 (H5C6 clone), PE-CD81 (5A6 clone), and PE-CD9 (HI9a clone) (BioLegend, San Diego, USA). The antibody master mix included the PanEV-marker, three disease-specific biomarkers, and the common leukocyte antigen CD45. The final mix consisted of 2 μL of PE-PanEV antibodies, 2 μL of Alexa Fluor^®^ 488 anti-Pan Cytokeratin (C-11, 10 μg/mL, BioLegend, San Diego, USA), 2.5 μL of Brilliant Violet 421™ anti-human CD45 (HI30, BioLegend, San Diego, USA), 2.5 μL of APC anti-PD-L1 (MIH1, Thermo Fisher Scientific, Massachusetts, USA), and 2.5 μL of APC-Cy7 Mouse Anti-Human CD326 (EpCAM, 9C4, BD Biosciences). Isotype-matched antibodies were used as negative controls. After incubation, the EV-EXÖBead complexes were washed twice with 1 mL of 0.5% EV-free BSA in PBS while retaining the magnetic beads using a magnet. The stained EV-EXÖBead complexes were then analyzed on a BD LSR Fortessa™ (BD Biosciences), and data processing was performed using FlowJo software (Tree Star, Ashland, Oregon, USA). Serum EV-EXÖBead complexes were first gated based on forward scatter (FSC-A) and side scatter (SSC-A) properties, corresponding to the 1 μm bead size. For double-positive EV-EXÖBead complex gating, populations were identified using the following criteria: PanEV+ and CD45+ or negative, PanEV+ and EpCAM+, PanEV+ and PD-L1+, and PanEV+ and PanCK+. These double-positive populations (PD-L1^+^ PanEV^+^, EpCAM^+^ PanEV^+^, PanCK^+^ PanEV^+^, CD45^+^ PanEV^+^, and CD45^−^ PanEV^+^) were gated based on background signal from unstained serum EV-EXÖBead complexes to ensure specificity, and for all patients and time points the final EV frequencies were calculated by subtracting the corresponding IgG isotype control frequency from the BEADS/Q2 double-positive population measured in the EV-stained samples.

### Nanoparticle tracking analysis

The concentration and size distribution of serum EVs were analyzed using the ZetaView^®^ Nanoparticle Tracking Analyzer PMX 110 (Particle Metrix GmbH), following the methodology described in our previous publication (35). EV samples were diluted in PBS to a final volume of 1 mL prior to measurement. Each analysis consisted of two measurement cycles, scanning 11 positions while capturing 60 frames per second. The pre-acquisition parameters were set with a sensitivity level of 80 and a shutter speed of 70, while the cell temperature was maintained at 25 °C and the trace length was set to 15. After data acquisition, video recordings were processed using ZetaView software version 8.05.11 SP1 (Particle Metrix GmbH). Analysis parameters included a minimum particle brightness threshold of 20, a particle size range of 5 to 1’000 pixels, a PSD nm/class value of 10, and PSD classes per decade set to 10.

### Statistical analysis

All statistical analyses were performed in RStudio (version: 2025.05.0 + 496) (38) with R (version: 4.5.1) (39) using the packages tidyverse, survival, survminer, ggplot2, ggpubr, stringr, purrr, grid, and RColorBrewer, with additional figure formatting in GraphPad Prism. Adjusted tab-delimited datasets were imported using read.delim, transposed so that patients represented rows and variables columns, and marker values were converted to numeric after removal of percent symbols where applicable; metadata variables, including smoking_status, OS_event, OS_time_months, EFS_event, and EFS_time_months, were formatted as factors or numeric variables as appropriate. Analyses were performed in both the full cohort and the current smoker subgroup. Pearson’s correlations were calculated between continuous EV marker levels and survival times (OS_time_months or EFS_time_months) using cor.test, and visualized with ggscatter (ggpubr) including regression lines, confidence intervals, and annotated correlation coefficients and p values. Exploratory Kaplan–Meier survival analyses were performed across TP1–TP5 for each marker root, with marker values dichotomized into high and low groups according to the median within each timepoint; survival curves for OS and EFS were estimated with survfit, group differences were assessed with survdiff, and figures were generated with ggsurvplot (survminer) using distinct TP-wise color palettes, with p values displayed for p < 0.1 and values < 0.05 highlighted in red. To avoid information loss from dichotomization and provide a more robust continuous-variable assessment, univariable Cox proportional hazards regression models were fitted separately for each marker and timepoint (TP1–TP5) for both OS and EFS using coxph and Surv from the survival package; marker values were standardized prior to analysis, and hazard ratios are reported per 1 standard deviation increase. Only patients with complete data for the respective marker and endpoint were included in each model. Model outputs included hazard ratios, 95% confidence intervals, p values, and Harrell’s concordance index (C-index). To reduce optimism related to the small sample size, an optimism-corrected Harrell’s C-index was estimated by bootstrap resampling (1,000 iterations), and results were exported as tab-separated files and visualized as forest-style plots with ggplot2; models were highlighted when they met both p < 0.1 and optimism-corrected C-index ≥ 0.75, with p < 0.05 shown in red and 0.05 ≤ p < 0.1 shown in black. Given the limited number of outcome events, no multivariable adjustment was performed, and all survival analyses were interpreted as exploratory rather than as evidence of independent prognostic value. To directly relate EV phenotypes to pathological response, additional non-parametric analyses were performed using MPR, pCR, and overall response to neoadjuvant chemotherapy as response variables. For these analyses, TP1–TP5 values were evaluated for PanEV, CD45, EpCAM, PDL1, PanCK, PDL1_PanEV, EpCAM_PanEV, PanCK_PanEV, CD45_PanEV, and CD45neg_PanEV. Group differences were assessed using the Wilcoxon rank-sum test for binary response variables and the Kruskal–Wallis test for variables with more than two categories. Significant associations (raw p < 0.05) were visualized using violin/boxplots with overlaid individual data points, and summary dot plots displaying −log10(p) were generated for significant marker-response associations only.

## Results

### Patients

Detailed characteristics of the trial population have been published (27). A cohort comprising 8 female and 12 male patients was selected from the trial SAKK 16/14 leftovers, for further characteristics also see **Table 1** (27). These patients were diagnosed with resectable stage IIIA(N2) NSCLC and exhibited a median overall survival of 3.22 years (see **Table 2**). The majority of patients, except for one, were either current or former smokers, with a median age of 61.6 years. Complete resection (R0) was achieved in all but one patient, who subsequently received adjuvant radiotherapy before starting adjuvant immunotherapy.

**Table 1:** Age and sex distribution in the cohort, tumor entity and T-Stadium at time of presentation.

Characteristics	Total (n=20)
Sex	
Female	8
Male	12
Age (years)	
Mean (Range)	61.6 (52 – 73)
T-Stadium at presentation (number)	
T1	5
T2	12
T3	3
Tumor entity	
Adenocarcinoma	13
Squamous cell carcinoma	5
NSCLC not otherwise specified	2
Smoking status (number)	
Current	11
Former	8
Never	1

**Table 2:** Key data: OS, EFS, MPR and pCR.

Overall survival (OS) - median	3.22 years
Event-free survival (EFS) - median	2.87 years
Major pathological response (MPR)	80%
Pathological complete response (pCR)	20%

### Isolation and quantification of extracellular vesicles

**Figure 1:**
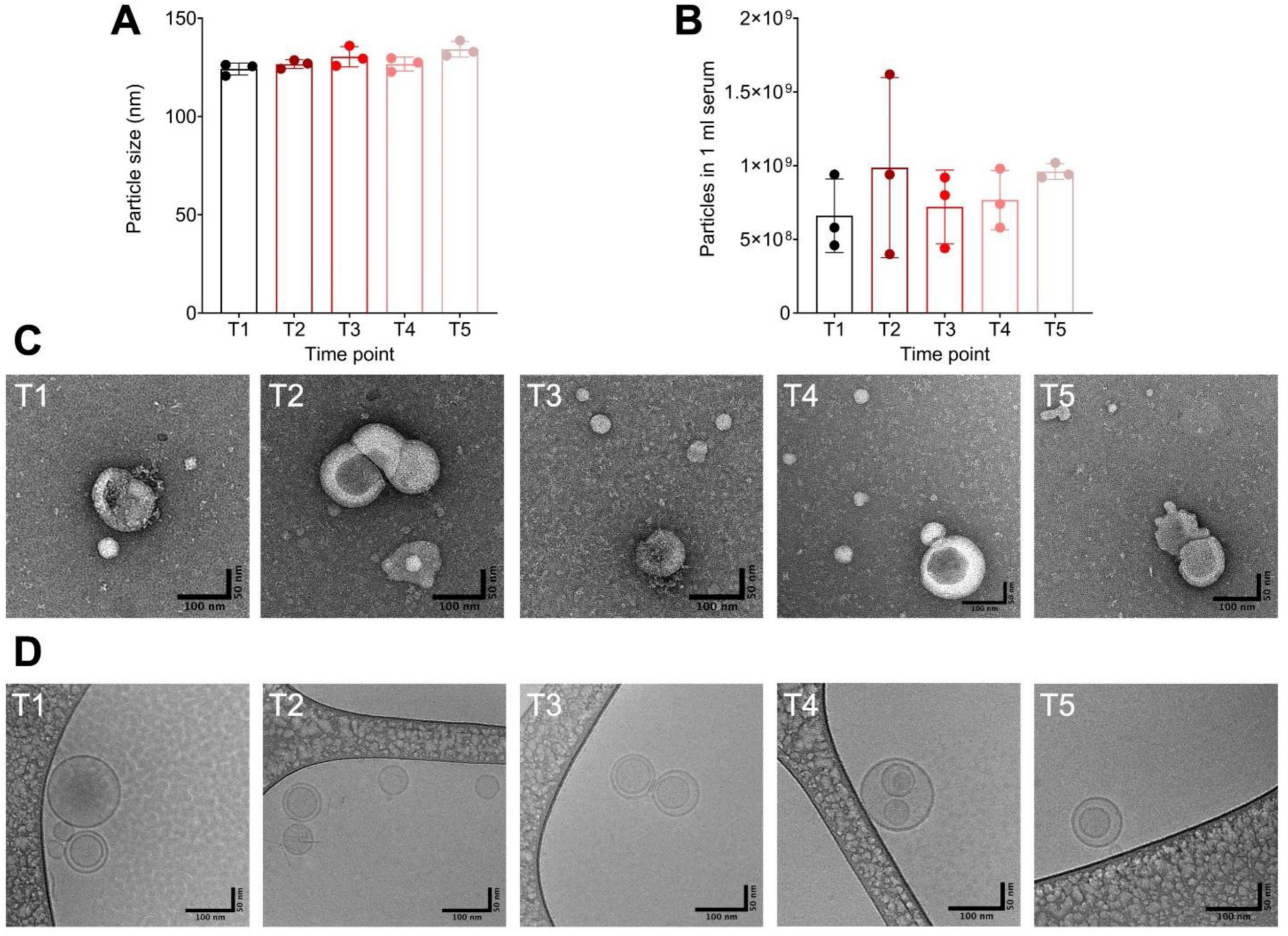
Size and concentration analysis of eluted extracellular vesicles (EVs). **(A)** Median particle size measured in three different patients at TP1–TP5. **(B)** Number of extracellular particles in 1 ml of serum across three different patients at TP1–TP5. **(C)** Negative-staining transmission electron microscopy of eluted EVs isolated by EXÖBead. **(D)** Cryo-electron microscopy.

EVs were successfully isolated from blood samples that had been stored for several years using our galectin-based EV isolation method. Specific EV markers were detected, confirming the identity of the isolated particles. As shown in **Figures 1A, B**, the particle sizes measured by nanoparticle tracking analysis (NTA) fall within the typical EV range of 30–150 nm, with the majority of particles around 150 nm. A bar chart summarizing the size distribution is provided in **Figure 1A**. The total particle concentration per 1 mL of serum is shown in **Figure 1B**, indicating that particle yield was within the expected range for serum-derived EVs. Furthermore, the morphology of the isolated vesicles was confirmed by transmission electron microscopy. Negative-staining electron microscopy (**Figure 1C**) revealed the characteristic cup-shaped appearance of EVs, while cryo-electron microscopy (cryo-EM) (**Figure 1D**) provided high-resolution visualization of their intact, lipid bilayer-enclosed structure under near-native conditions. Notably, no significant changes in particle size or concentration were observed over the course of treatment. In conclusion, our isolation approach fulfills the internationally accepted minimal requirements for EV isolation as defined by the MISEV 2023 guidelines (40).

### Prognostic potential

We sought to quantify changes in EV levels in patients undergoing cancer treatment to evaluate their potential as prognostic biomarkers. To assess the prognostic significance of EV-associated markers, patients were stratified into two groups based on the median gating values, resulting in a high-expression group (above the mean) and a low-expression group (below the mean).

**Figure 2:**
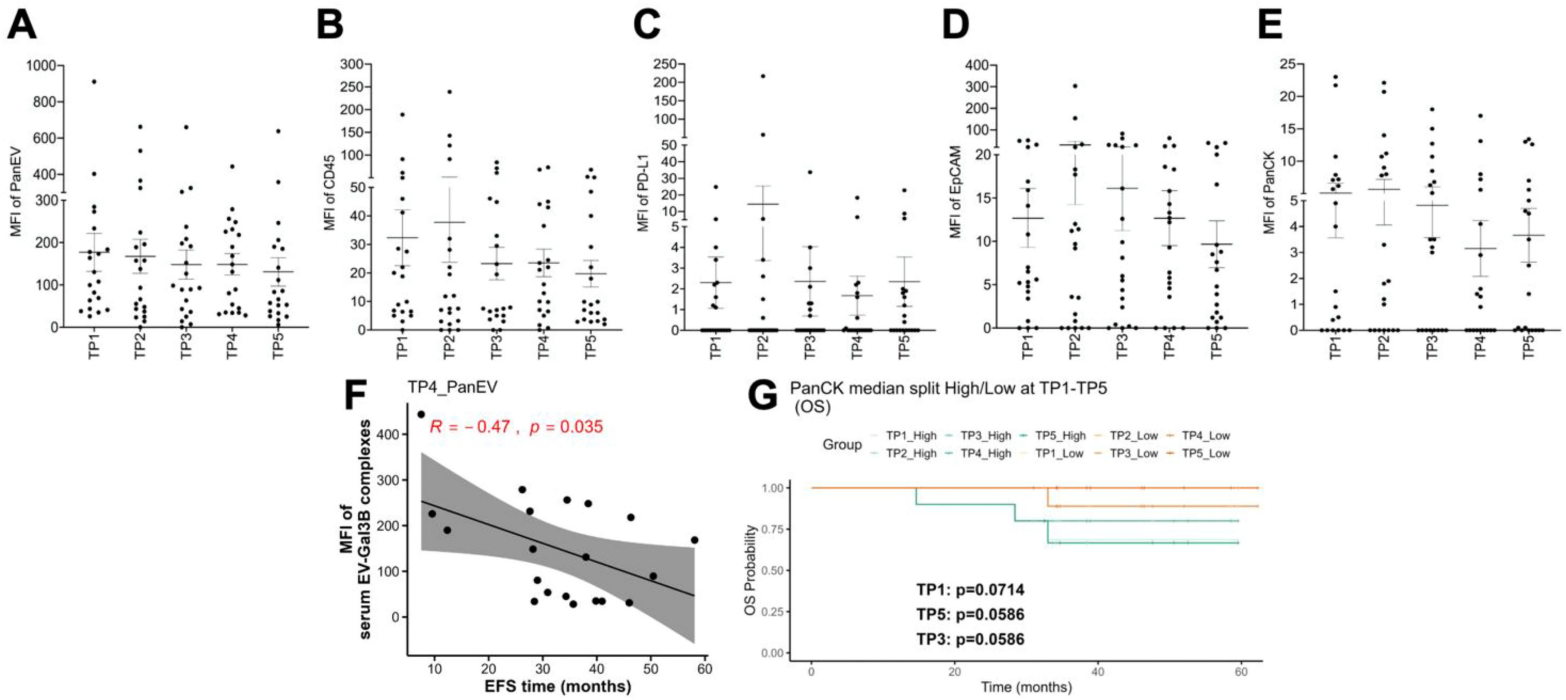
**(A–E)** Mean fluorescence intensity (MFI) of different surface markers across all patients and time points (TP1–TP5). **(F)** Correlation analysis of PanCK^+^-EXÖBead complex at TP4 with EFS (p = 0.035). **(G)** Kaplan–Meier analysis of OS based on PanCK^+^-EXÖBead complex at TP1–5 for high and low MFI values at individual time points.

The mean fluorescence intensity (MFI) values for all patients across all time points are presented in **Figure 2**. Multivariate ANOVA analysis revealed no statistically significant differences between time points. Nevertheless, a downward trend in MFI values was observed following the first treatment, particularly for the PD-L1 and EpCAM markers (**Figures 2C, D**). Substantial inter-patient variability was evident, with a considerable subset of patients exhibiting low MFI values. Correlation analysis of PanEV MFI at TP4 with event-free survival (EFS) demonstrated a negative association (p = 0.035; **Figure 2F**). Furthermore, PanCK MFI values at different time points were analyzed in relation to OS using Kaplan–Meier curves. As shown in **Figure 2G**, a trend toward worse OS was observed in the high-expression group compared with the low-expression group, although this difference did not reach statistical significance.

**Figure 3:**
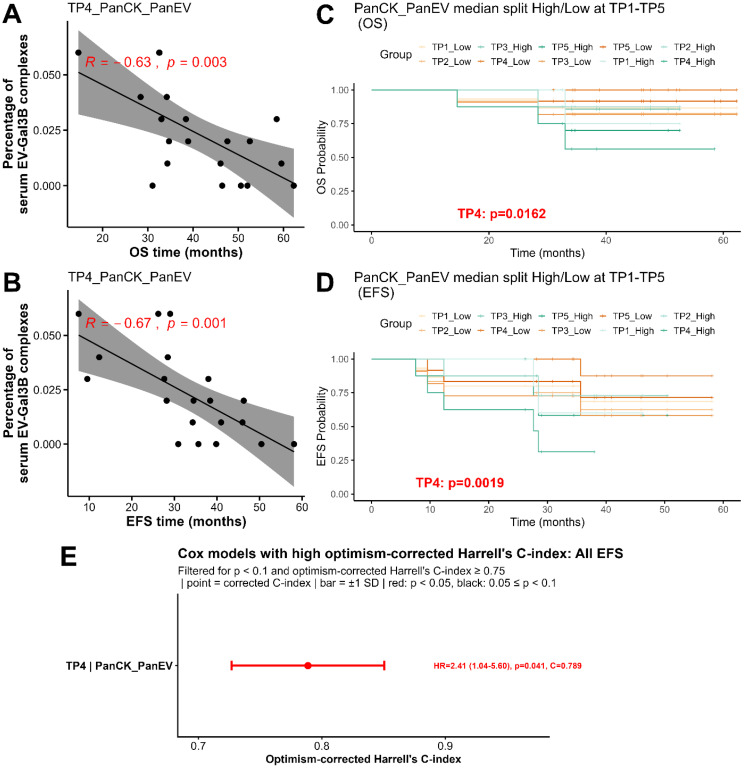
**(A, B)** Correlation of PanCK^+^/PanEV^+^-EXÖBead complex at TP4 with OS (p = 0.003) and EFS (p = 0.001). **(C, D)** Kaplan–Meier analysis of OS and EFS stratified by TP4 PanCK^+^/PanEV^+^-EXÖBead levels (OS: p = 0.0162; EFS: p = 0.0019). **(E)** Continuous-variable Cox regression for EFS at TP4 for PanCK^+^ PanEV^+^ EV-EXÖBead complex (HR per 1 SD: 2.41, 95% CI 1.04–5.60, p = 0.041, optimism-corrected C-index = 0.789); points indicate corrected C-index and bars ±1 SD from bootstrap resampling.

The further analyses focused on PanCK^+^/PanEV^+^-double-positive EV levels at different time points, correlating these findings with clinical outcomes such as OS and EFS. As shown in **Figures 3A, B**, PanCK^+^/PanEV^+^-EXÖBead complex levels at TP4 were significantly correlated with survival outcomes, with p-values of 0.003 for OS and 0.001 for EFS. Consistent findings were obtained by Kaplan–Meier analyses, where high expression of the PanCK^+^/PanEV^+^-EXÖBead complex at TP4 was associated with significantly poorer OS (p = 0.0162) and EFS (p = 0.0019) (**Figures 3C, D**). Similar trends were observed at different time points, although statistical significance was not reached. Additionally, in continuous-variable Cox regression analysis of event-free survival, higher levels of PanCK^+^/PanEV^+^ EV-EXÖBead complexes at TP4 were significantly associated with an increased risk of events, with a hazard ratio of 2.41 per 1 standard deviation increase (95% CI 1.04–5.60, p = 0.041). Model discrimination was internally validated using bootstrap resampling (1,000 iterations), yielding an optimism-corrected Harrell’s C-index of 0.789, indicating robust discriminatory performance after adjustment for optimism. (**Figure 3E**).

Analysis of EpCAM^+^/PanEV^+^ double-positive complexes at TP4 via Kaplan-Meier curves revealed a trend toward reduced OS in the high-expression group especially after treatment at TP 4 (p = 0.059), as illustrated in **Supplementary Figure 2**.

### Extracellular vesicles in smokers

**Figure 4:**
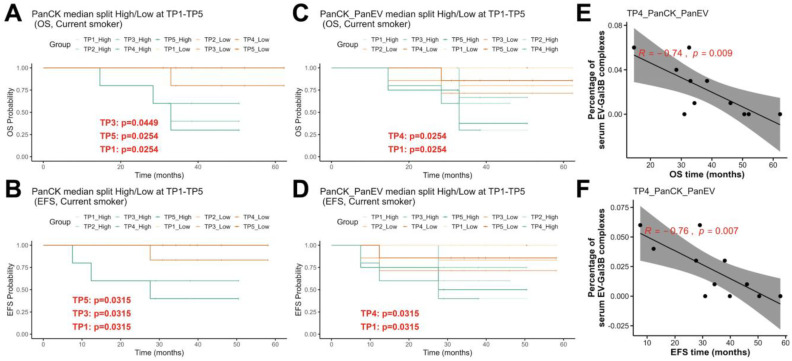
Prognostic significance of EV-associated markers in active smokers. **(A, B)** Kaplan–Meier survival analysis of the PanCK^+^-EXÖBead complex at TP1–5 for EFS and OS. **(C, D)**. Kaplan–Meier survival analysis of the PanCK^+^/PanEV^+^-EXÖBead complex at TP1–5 for EFS and OS. **(E, F)** Correlation of PanCK^+^/PanEV^+^ signal on the EV-EXÖBead complex to OS (p = 0.009) and EFS (p = 0.007) at TP4.

To evaluate the impact of EV levels in the blood of active smokers, we analyzed the samples from the 11 patients in the current smoker group to assess whether EV-associated markers contribute to disease progression. Here we focused on the analysis of PanCK^+^ EVs. As shown in **Figure 4A**, patients with high PanCK^+^-EXÖBead complex expression exhibited a significantly reduced OS compared to those with lower expression levels (p = 0.0449 for TP3 and 0.0254 for TP5 and TP1). Similar trends were observed for EFS, with p-values of 0.0315 at TP1, TP3 and TP5. Further analyses using double-positive PanCK^+^/PanEV^+^-EXÖBead complexes yielded consistent results (**Figures 3C, D**). Correlation analyses of PanCK^+^/PanEV^+^-EXÖBead complex levels at TP4 demonstrated strong negative correlation with OS (R = -0.74, p = 0.009) and for EFS (R = -0.76, p = 0.007), indicating a robust association with poor outcomes.

In addition, analysis of PD-L1^+^/PanEV^+^ marker levels at TP1 revealed significantly higher values in patients achieving pCR compared with non-pCR patients (Wilcoxon p = 0.038), suggesting that elevated circulating PD-L1^+^ EV levels may be linked to improved treatment response in current smokers (**Supplementary Figure 5**).

Mean fluorescence intensity (MFI) of PanCK- and PanEV-EXÖBead complexes at TP4 also significantly correlated with EFS, supporting their potential utility as prognostic markers. In addition, correlation analyses of both CD45^-^ -and CD45^+^/PanEV^+^-EXÖBead complexes showed statistically significant associations (p <0.05). (**Supplementary Figure 3**). Kaplan-Meier survival analyses for CD45^-^ -and CD45^+^/PanEV^+^-EXÖBead complexes did not show significant results.

Further Kaplan-Meier survival analyses for PanCK, CD45, EpCAM, PanCK and also PanEV at various time points, highlighting statistically significant results, are presented in **Supplementary Figure 4**.

## Discussion

The trial SAKK 16/14 (27) was one of the first studies showing that the addition of perioperative immune checkpoint inhibition to neoadjuvant chemotherapy in patients with resectable stage IIIA NSCLC with confirmed mediastinal lymph node involvement is feasible and improves outcome compared to standard neoadjuvant chemotherapy alone. In this exploratory study, we analyzed serum samples from 20 selected patients to investigate EV biomarkers and their clinical relevance. Our goal of finding a correlation of changes in EV protein expression with therapy progress and clinical outcomes, particularly OS and EFS was achieved. Additionally, in the PanCK^+^/PanEV^+^ EXÖBead complex we found a marker with promising prognostic potential.

A major strength of this study was the availability of serial blood samples collected at defined therapy time points, enabling longitudinal profiling. Using our galectin-coupled, magnetic bead-based method, EXÖBead (41), as a liquid biopsy biomarker we were able to analyze small samples, as little as 1 ml of serum, even after prolonged sample storage for several years. As shown in our previous publication (35), EXÖBead efficiently isolates EVs expressing established markers (CD9, CD63, CD81) with low lipoprotein contamination and preserved EV functionality. Owing to its glycan-binding capture mechanism, EXÖBead likely enriches glycan/glycoprotein-bearing EVs and may therefore preferentially represent specific circulating EV subpopulations rather than the entire EV pool.

Our data indicate that dynamic changes in EV expression are associated with clinical outcomes. Pronounced results were seen in PanEV analysis in general, both in single-parameter assessments and in double-positive marker combinations. Posttherapeutically, when tumor burden is expected to be lowest, corresponding in our cohort to TP4, a reduction in circulating PanEV levels was anticipated. This expectation is biologically plausible, as tumor cells constitutively release EVs, and both their abundance and molecular cargo reflect the biological state of the cell of origin. In addition, EV biogenesis may be enhanced by microenvironmental stressors such as hypoxia, which commonly increases with tumor burden (42, 43). Additionally, effective therapy may also induce an initial transient increase in apoptotic vesicle release from dying tumor cells, followed by an overall decline in tumor-associated EV signals as viable tumor mass decreases (44, 45). Supporting this concept, Peng et al. (46) reported a significant post-immunotherapy decrease in plasma hsa-miR-125b-5p levels in patients achieving a partial response. In line with this rationale are our findings, as patients exhibiting elevated PanEV levels at TP4 showed significantly shorter EFS, as illustrated in **Figure 2F**. Collectively, these findings are consistent with previous reports demonstrating that systemic therapy can modulate circulating EV profiles in a manner detectable by liquid biopsy approaches.

Single-marker analysis of EpCAM, PanCK, PD-L1, and CD45, as depicted in **Figures 2B–E**, revealed trends suggestive of clinical utility, highlighting their potential role in longitudinal therapy monitoring in NSCLC. Notably, our data reinforces the previously proposed association between reduced CD45 expression and adverse clinical outcome, as described by Ye et al. (34) in 2022. These additional markers help broadening the horizon of possible markers that can also be applied in EV analysis of NSCLC probes.

Especially noteworthy is the extended analysis of PanCK^+^/PanEV^+^ double-positive EVs at TP4, demonstrating statistically significant association with both diminished OS and EFS, yielding p-values of 0.003 and 0.001, respectively, in the direct correlation (**Figure 3**). In the Kaplan-Meier analysis p-values of 0.0162 for OS and 0.0019 for EFS were calculated. The optimism-corrected Harrell’s C-Index of approximately 0.8 for EFS corroborates the previous mentioned findings for PanCK^+^/PanEV^+^-EXÖBead complex at TP4. Collectively, these findings suggest the integration of EV profiling as a prognostic biomarker strategy and adjunct tool for therapeutic response assessment in patients undergoing multimodal cancer treatment. In this context, such biomarkers may, in future studies, contribute to more individualized treatment strategies, including the potential intensification of adjuvant therapy in patients identified as having an adverse prognosis. The availability of an additional tool to assess therapy response is particularly valuable in cases of unconventional response patterns, such as those observed during immunotherapy, where distinguishing between pseudo-progression and true tumor progression can be challenging. However, it should be noted that PanCK positivity is not exclusively tumor-specific, as cytokeratin-positive EVs may also originate from non-malignant epithelial cells undergoing physiological turnover, inflammation, or treatment-induced tissue damage (47, 48). While PanCK^+^/PanEV^+^ EVs appear to reflect tumor-derived vesicles in the studied context further investigation is needed to more precisely determine their cellular origin and to distinguish tumor-derived EVs from those released by non-malignant epithelial cells under physiological or treatment-related conditions.

Several studies have investigated the effect of cigarette smoke on EV biogenesis and release, contributing to chronic lung disease and lung cancer, although the underlying mechanisms remain incompletely understood (49–51). In our study, we were able to reproduce these findings, showing that smoking affects EV levels pre- and intra-therapeutic. Notably, PanCK^+^ single-positive and double-positive PanCK^+^/PanEV^+^-EXÖBead complexes in smokers were significantly associated with both OS and EFS (**Figure 4**). While EFS was comparable between the full cohort and the smoker subgroup, OS was significantly poorer among smokers with elevated EV levels, with approximately 70% mortality at 40 months compared to 50% in the overall cohort, in line with established clinical expectations regarding smoking-related risk. Importantly, significant associations were observed in both smokers and the full cohort, supporting the potential of the PanCK^+^/PanEV^+^-EXÖBead complex as a prognostic marker independent of smoking status. At TP4, PanEV and PanCK MFI showed prognostic relevance (p = 0.046 and p = 0.017, respectively), and CD45^−^ and CD45^+^/PanEV^+^-EXÖBead complex levels were inversely correlated with OS and EFS (p < 0.05) (**Supplementary Figure 3**), although Kaplan–Meier analyses did not reach statistical significance, likely due to limited cohort size. Taken together, these findings highlight the importance of biomarker selection tailored to clinical variables such as smoking status and sampling time point, supporting personalized medicine strategies. While these results are compelling, they must be interpreted in the context of our limited sample size and the absence of precise quantitative smoking data. Furthermore, flow cytometry was performed on EV-EXÖBead complexes rather than single EVs or particle-count-normalized EV preparations. Thus, the reported MFI reflects the overall signal intensity of EVs captured on EXÖBeads rather than marker expression per individual vesicle. As normalization to EV particle counts was not performed, variability in EV yield or capture efficiency between samples may have influenced measured marker intensities.

We anticipated that PD-L1 levels in EVs would correlate with clinical data, as described by Li et al. (24), who demonstrated that exo-PD-L1 levels were positively associated with clinicopathologic disease indicators, including tumor size, lymph node status, distant metastasis, and TNM stage. The value of exosomal PD-L1 in diagnostics and clinical potential has also been described and evaluated before, for example by Liu et al. (52), Peng et al. (53) and Theodoraki et al. (14) Although PD-L1 was detectable in circulating EVs of NSCLC patients - consistent with prior findings (11, 54) - its expression levels were generally low and did not demonstrate clear prognostic or predictive significance in the overall cohort. However, in the subgroup of current smokers, elevated PD-L1^+^/PanEV^+^ EV levels at TP1 were significantly associated with achievement of pCR, suggesting that EV-associated PD-L1 may identify patients with enhanced sensitivity to multimodal treatment including durvalumab. (**Supplementary Figure 5**). This observation is biologically plausible and consistent with prior clinical evidence demonstrating improved efficacy of durvalumab in tumors with higher PD-L1 expression. In the PACIFIC trial, progression-free and overall survival benefit increased with rising tumor PD-L1 expression, whereas patients with PD-L1 <1% derived no clear overall survival benefit in exploratory subgroup analyses (55). Further studies supported PD-L1 as a predictive biomarker for durvalumab efficacy, showing superior outcomes in patients with PD-L1-positive tumors and limited benefit in PD-L1-negative disease (56, 57). The discrepancy between the full cohort and the smoking subgroup may reflect biological heterogeneity, limited sample size, or differential immune activation associated with tobacco exposure, and should be investigated in dedicated, larger prospective studies.

While our study provides insights into the potential of EVs, several limitations should be acknowledged. Firstly, the relatively small cohort size constrains statistical power and may limit extrapolation to larger populations. Future studies should aim to include larger and more diverse cohorts to validate and extend our findings. Secondly, the primary study was not designed for the purpose of our analysis and may be understood as an exploratory study. And thirdly, for our study we received serum samples instead of plasma. As described before by Zhang et al. (58) serum may contain additional extracellular vesicles deriving from platelets during clot formation, which can affect EV characterization both quantitatively and qualitatively. Specifically, serum shows higher total particle counts by nanoparticle tracking analysis and selective enrichment of platelet-associated proteins, which confers different proteome profiles compared to plasma EVs. The extent to which platelet-derived EVs may have influenced our measurements is not quantified. Therefore, this pre-analytical variable should be considered when interpreting our findings and when comparing them with studies using plasma samples (59, 60).

As strong point, we want to highlight the fact that comprehensive clinical and survival data from the trial SAKK 16/14 was available and used for our analysis. Additionally, a correlation of our measurements with EFS and OS is possible and statistically significant. And finally, our study represents one of the first efforts to deliver significant data analyzing the effects of different therapy modalities including chemo- and immunotherapy and surgery on EV markers in patients with NSCLC.

In conclusion we state that EVs have a considerable potential to be a valuable clinical asset in diagnostics, prognosis, therapy-response-monitoring and even recurrence monitoring in the follow-up in cancer patients. EV profiling in liquid biopsies offers a simple, minimally invasive and highly informative approach that may complement existing diagnostic modalities. The clinical value of such biomarker strategies is expected to be particularly pronounced in highly heterogeneous disease settings, such as resectable stage IIIA (N2) NSCLC, where individualized treatment decisions are critical. The insights gained here not only advance the understanding of EV biology in lung cancer but may also be translatable to other malignancies, such as head and neck cancer (41). Continued investigation in larger, prospective trials is needed to validate the potential and possible limitations of EV biomarkers in clinical practice.

## Data Availability

The datasets presented in this study can be found in online repositories. The names of the repository/repositories and accession number(s) can be found in the article/**Supplementary Material**.

## Supplementary material

Supplementary Figure 1 Trial design and extracellular vesicle isolation workflow. Trial design adapted from Rothschild, Sacha I., et al. “SAKK 16/14: durvalumab in addition to neoadjuvant chemotherapy in patients with stage IIIA (N2) non–small-cell lung cancer—a multicenter single-arm phase II trial.” **(a)** Workflow of extracellular vesicle (EV) isolation and characterization adapted from Benecke, Laura et al. “Isolation and analysis of tumor−derived extracellular vesicles from head and neck squamous cell carcinoma plasma by galectin−based glycan recognition particles.” Created in BioRender. Chiang, M. (2025) https://BioRender.com/7sfvuh0 **(b)**.
