# Supplementary material for: Extracellular vesicles as prognostic biomarkers: results of a neoadjuvant chemoimmunotherapy clinical trial in stage IIIA (N2) non-small-cell lung cancer (SAKK 16/14)
Source: Front Immunol. 2026 Jul 1;17:1807542. doi: 10.3389/fimmu.2026.1807542 (PMC13369264; doi:10.3389/fimmu.2026.1807542)
Supplement: Supplementary Figure 1 — Trial design and extracellular vesicle isolation workflow. Trial design adapted from Rothschild, Sacha I., et al. “SAKK 16/14: durvalumab in addition to neoadjuvant chemotherapy in patients with stage IIIA (N2) non–small-cell lung cancer—a multicenter single-arm phase II trial.” (a) Workflow of extracellular vesicle (EV) isolation and characterization adapted from Benecke, Laura et al. “Isolation and analysis of tumor−derived extracellular vesicles from head and neck squamous cell carcinoma plasma by galectin−based glycan recognition particles.” Created in BioRender. Chiang, M. (2025) https://BioRender.com/7sfvuh0 (b). [file DataSheet1.zip › Gated_Raw_flow_data/(003 + 004) MFI.pdf]

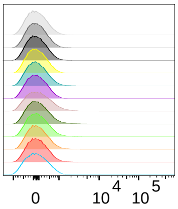

PE-A :: PanEV

Sample Name	Median : PE-A	Mean : PE-A	Geometric Mean : PE-A
Specimen_001_004_TP5_1 ml_EV staining_012.fcs	8.97	68.2	31.6
Specimen_001_004_TP4_1 ml_EV staining_011.fcs	8.97	64.1	29.5
Specimen_001_004_TP3_1 ml_EV staining_010.fcs	8.97	66.1	30.3
Specimen_001_004_TP2_1 ml_EV staining_009.fcs	10.5	78.3	30.2
Specimen_001_004_TP1_1 ml_EV staining_008.fcs	15.0	73.4	39.3
Specimen_001_004_TP1-5_total_1 ml_IgG_007.fcs	10.5	29.7	24.2
Specimen_001_003_TP5_1 ml_EV staining_006.fcs	71.9	277	122
Specimen_001_003_TP4_1 ml_EV staining_005.fcs	46.4	200	87.6
Specimen_001_003_TP3_1 ml_EV staining_004.fcs	43.4	127	74.7
Specimen_001_003_TP2_1 ml_EV staining_003.fcs	40.4	123	71.2
Specimen_001_003_TP1_1 ml_EV staining_002.fcs	35.9	92.0	58.6
Specimen_001_003_TP1-5_total_1 ml_IgG_001.fcs	5.98	28.5	21.1

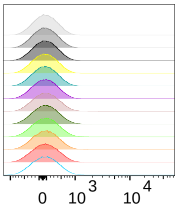

BV421-A :: CD45

Sample Name	Median : BV421-A	Mean : BV421-A	Geometric Mean : BV421-A
Specimen_001_004_TP5_1 ml_EV staining_012.fcs	75.3	81.8	75.5
Specimen_001_004_TP4_1 ml_EV staining_011.fcs	69.9	74.8	69.7
Specimen_001_004_TP3_1 ml_EV staining_010.fcs	73.2	77.5	72.4
Specimen_001_004_TP2_1 ml_EV staining_009.fcs	67.7	73.5	67.4
Specimen_001_004_TP1_1 ml_EV staining_008.fcs	67.7	72.8	67.6
Specimen_001_004_TP1-5_total_1 ml_IgG_007.fcs	74.2	78.1	72.7
Specimen_001_003_TP5_1 ml_EV staining_006.fcs	94.0	131	105
Specimen_001_003_TP4_1 ml_EV staining_005.fcs	87.4	106	92.4
Specimen_001_003_TP3_1 ml_EV staining_004.fcs	89.6	101	90.6
Specimen_001_003_TP2_1 ml_EV staining_003.fcs	87.4	102	89.3
Specimen_001_003_TP1_1 ml_EV staining_002.fcs	76.4	88.0	78.5
Specimen_001_003_TP1-5_total_1 ml_IgG_001.fcs	74.2	81.8	73.8

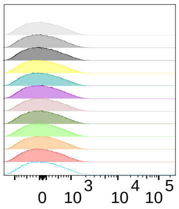

APC-Cy7-A :: EpCAM

Sample Name	Median : APC-Cy7-A	Mean : APC-Cy7-A	Geometric Mean : APC-Cy7-A
Specimen_001_004_TP5_1 ml_EV staining_012.fcs	-16.7	18.2	7.37
Specimen_001_004_TP4_1 ml_EV staining_011.fcs	-30.8	5.92	-2.68
Specimen_001_004_TP3_1 ml_EV staining_010.fcs	-32.1	7.91	-3.51
Specimen_001_004_TP2_1 ml_EV staining_009.fcs	-30.8	11.0	-1.58
Specimen_001_004_TP1_1 ml_EV staining_008.fcs	-25.7	10.4	0.31
Specimen_001_004_TP1-5_total_1 ml_IgG_007.fcs	-24.4	8.78	-0.20
Specimen_001_003_TP5_1 ml_EV staining_006.fcs	2.57	46.1	28.3
Specimen_001_003_TP4_1 ml_EV staining_005.fcs	-18.0	26.1	7.62
Specimen_001_003_TP3_1 ml_EV staining_004.fcs	-8.98	26.6	15.7
Specimen_001_003_TP2_1 ml_EV staining_003.fcs	-3.85	33.6	19.1
Specimen_001_003_TP1_1 ml_EV staining_002.fcs	-24.4	13.1	3.35
Specimen_001_003_TP1-5_total_1 ml_IgG_001.fcs	-1.07E-13	39.2	22.7

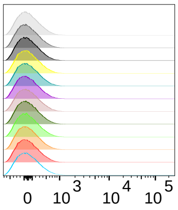

APC-A :: PD-L1

Sample Name	Median : APC-A	Mean : APC-A	Geometric Mean : APC-A
Specimen_001_004_TP5_1 ml_EV staining_012.fcs	-5.36	22.7	18.3
Specimen_001_004_TP4_1 ml_EV staining_011.fcs	-13.9	14.0	10.4
Specimen_001_004_TP3_1 ml_EV staining_010.fcs	-9.64	18.1	13.9
Specimen_001_004_TP2_1 ml_EV staining_009.fcs	-7.50	19.6	15.4
Specimen_001_004_TP1_1 ml_EV staining_008.fcs	-8.57	19.3	15.3
Specimen_001_004_TP1-5_total_1 ml_IgG_007.fcs	-13.9	14.1	10.1
Specimen_001_003_TP5_1 ml_EV staining_006.fcs	-8.57	19.3	14.7
Specimen_001_003_TP4_1 ml_EV staining_005.fcs	-9.64	19.0	14.7
Specimen_001_003_TP3_1 ml_EV staining_004.fcs	-12.9	16.2	12.4
Specimen_001_003_TP2_1 ml_EV staining_003.fcs	-11.8	15.5	11.7
Specimen_001_003_TP1_1 ml_EV staining_002.fcs	-10.7	16.4	12.6
Specimen_001_003_TP1-5_total_1 ml_IgG_001.fcs	-12.9	17.3	10.7

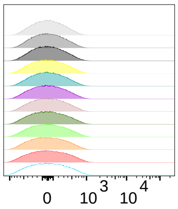

FITC-A :: PanCK

Sample Name	Median : FITC-A	Mean : FITC-A	Geometric Mean : FITC-A
Specimen_001_004_TP5_1 ml_EV staining_012.fcs	9.19	16.8	13.6
Specimen_001_004_TP4_1 ml_EV staining_011.fcs	9.19	17.8	14.3
Specimen_001_004_TP3_1 ml_EV staining_010.fcs	7.66	15.4	12.2
Specimen_001_004_TP2_1 ml_EV staining_009.fcs	5.36	13.4	10.0
Specimen_001_004_TP1_1 ml_EV staining_008.fcs	7.66	16.1	12.7
Specimen_001_004_TP1-5_total_1 ml_IgG_007.fcs	4.59	12.2	9.19
Specimen_001_003_TP5_1 ml_EV staining_006.fcs	9.19	17.4	13.2
Specimen_001_003_TP4_1 ml_EV staining_005.fcs	4.59	13.7	9.75
Specimen_001_003_TP3_1 ml_EV staining_004.fcs	3.06	10.7	7.69
Specimen_001_003_TP2_1 ml_EV staining_003.fcs	3.83	15.1	8.73
Specimen_001_003_TP1_1 ml_EV staining_002.fcs	9.19	18.7	14.0
Specimen_001_003_TP1-5_total_1 ml_IgG_001.fcs	9.95	17.2	12.9
