# Supplementary material for: Extracellular vesicles as prognostic biomarkers: results of a neoadjuvant chemoimmunotherapy clinical trial in stage IIIA (N2) non-small-cell lung cancer (SAKK 16/14)
Source: Front Immunol. 2026 Jul 1;17:1807542. doi: 10.3389/fimmu.2026.1807542 (PMC13369264; doi:10.3389/fimmu.2026.1807542)
Supplement: Supplementary Figure 1 — Trial design and extracellular vesicle isolation workflow. Trial design adapted from Rothschild, Sacha I., et al. “SAKK 16/14: durvalumab in addition to neoadjuvant chemotherapy in patients with stage IIIA (N2) non–small-cell lung cancer—a multicenter single-arm phase II trial.” (a) Workflow of extracellular vesicle (EV) isolation and characterization adapted from Benecke, Laura et al. “Isolation and analysis of tumor−derived extracellular vesicles from head and neck squamous cell carcinoma plasma by galectin−based glycan recognition particles.” Created in BioRender. Chiang, M. (2025) https://BioRender.com/7sfvuh0 (b). [file DataSheet1.zip › Gated_Raw_flow_data/(008 + 066) MFI.pdf]

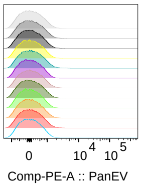

Sample Name	Median : Comp-PE-A	Mean : Comp-PE-A	Geometric Mean : Comp-PE-A
Specimen_001_066 TP5 1 ml serum+ 900 UL PBS (10000g)_006.fcs	61.4	114	80.8
Specimen_001_066 TP4 1 ml serum+ 900 UL PBS (10000g)_005.fcs	55.4	107	75.1
Specimen_001_066 TP3 1 ml serum+ 900 UL PBS (10000g)_004.fcs	44.9	85.9	61.0
Specimen_001_066 TP2 1 ml serum+ 900 UL PBS (10000g)_003.fcs	44.9	82.2	58.7
Specimen_001_066 TP1 1 ml serum+ 900 UL PBS (10000g)_002.fcs	77.9	146	98.6
Specimen_001_066 (200ul x5)+ 900 UL PBS (IgG)_001.fcs	31.4	58.2	44.1
Specimen_001_008 TP5 1 ml serum+ 900 UL PBS (10000g)_011.fcs	64.4	124	85.3
Specimen_001_008 TP4 1 ml serum+ 900 UL PBS (10000g)_010.fcs	38.9	83.9	56.2
Specimen_001_008 TP3 1 ml serum+ 900 UL PBS (10000g)_009.fcs	28.4	62.7	43.7
Specimen_001_008 TP2 1 ml serum+ 900 UL PBS (10000g)_008.fcs	34.4	71.1	52.0
Specimen_001_008 TP1 1 ml serum+ 900 UL PBS (10000g)_007.fcs	50.9	93.1	66.9
Specimen_001_008 (200 ul x5)+ 900 ul PBS (IgG)_012.fcs	28.4	57.1	43.5

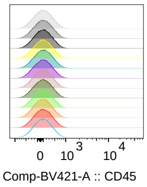

Sample Name	Median : Comp-BV421-A	Mean : Comp-BV421-A	Geometric Mean : Comp-BV421-A
Specimen_001_066 TP5 1 ml serum+ 900 UL PBS (10000g)_006.fcs	101	107	98.8
Specimen_001_066 TP4 1 ml serum+ 900 UL PBS (10000g)_005.fcs	103	109	102
Specimen_001_066 TP3 1 ml serum+ 900 UL PBS (10000g)_004.fcs	98.3	102	95.9
Specimen_001_066 TP2 1 ml serum+ 900 UL PBS (10000g)_003.fcs	98.3	102	96.1
Specimen_001_066 TP1 1 ml serum+ 900 UL PBS (10000g)_002.fcs	115	122	114
Specimen_001_066 (200ul x5)+ 900 UL PBS (IgG)_001.fcs	97.2	99.2	94.0
Specimen_001_008 TP5 1 ml serum+ 900 UL PBS (10000g)_011.fcs	98.3	105	96.2
Specimen_001_008 TP4 1 ml serum+ 900 UL PBS (10000g)_010.fcs	95.1	103	92.8
Specimen_001_008 TP3 1 ml serum+ 900 UL PBS (10000g)_009.fcs	95.1	101	93.2
Specimen_001_008 TP2 1 ml serum+ 900 UL PBS (10000g)_008.fcs	90.7	94.9	89.4
Specimen_001_008 TP1 1 ml serum+ 900 UL PBS (10000g)_007.fcs	98.3	104	96.9
Specimen_001_008 (200 ul x5)+ 900 ul PBS (IgG)_012.fcs	95.1	97.1	92.3

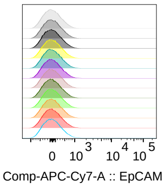

Sample Name	Median : Comp-APC-Cy7-A	Mean : Comp-APC-Cy7-A	Geometric Mean : Comp-APC-Cy7-A
Specimen_001_066 TP5 1 ml serum+ 900 UL PBS (10000g)_006.fcs	-16.7	5.14	1.65
Specimen_001_066 TP4 1 ml serum+ 900 UL PBS (10000g)_005.fcs	-11.6	7.11	4.50
Specimen_001_066 TP3 1 ml serum+ 900 UL PBS (10000g)_004.fcs	-15.4	3.14	0.88
Specimen_001_066 TP2 1 ml serum+ 900 UL PBS (10000g)_003.fcs	-10.3	10.8	6.06
Specimen_001_066 TP1 1 ml serum+ 900 UL PBS (10000g)_002.fcs	-14.1	5.43	3.14
Specimen_001_066 (200ul x5)+ 900 UL PBS (IgG)_001.fcs	-16.7	0.86	-1.11
Specimen_001_008 TP5 1 ml serum+ 900 UL PBS (10000g)_011.fcs	-11.6	8.52	4.13
Specimen_001_008 TP4 1 ml serum+ 900 UL PBS (10000g)_010.fcs	-14.1	14.1	2.50
Specimen_001_008 TP3 1 ml serum+ 900 UL PBS (10000g)_009.fcs	-11.6	12.9	5.29
Specimen_001_008 TP2 1 ml serum+ 900 UL PBS (10000g)_008.fcs	-14.1	3.41	1.06
Specimen_001_008 TP1 1 ml serum+ 900 UL PBS (10000g)_007.fcs	-14.1	6.53	3.23
Specimen_001_008 (200 ul x5)+ 900 ul PBS (IgG)_012.fcs	-18.0	-0.093	-2.02

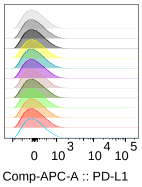

Sample Name	Median : Comp-APC-A	Mean : Comp-APC-A	Geometric Mean : Comp-APC-A
Specimen_001_066 TP5 1 ml serum+ 900 UL PBS (10000g)_006.fcs	-11.6	28.7	21.6
Specimen_001_066 TP4 1 ml serum+ 900 UL PBS (10000g)_005.fcs	-14.1	26.7	19.4
Specimen_001_066 TP3 1 ml serum+ 900 UL PBS (10000g)_004.fcs	-11.6	27.4	20.3
Specimen_001_066 TP2 1 ml serum+ 900 UL PBS (10000g)_003.fcs	-12.8	27.6	19.8
Specimen_001_066 TP1 1 ml serum+ 900 UL PBS (10000g)_002.fcs	-18.0	22.5	15.7
Specimen_001_066 (200ul x5)+ 900 UL PBS (IgG)_001.fcs	-15.4	26.9	19.7
Specimen_001_008 TP5 1 ml serum+ 900 UL PBS (10000g)_011.fcs	-15.4	28.4	20.7
Specimen_001_008 TP4 1 ml serum+ 900 UL PBS (10000g)_010.fcs	-15.4	29.3	19.0
Specimen_001_008 TP3 1 ml serum+ 900 UL PBS (10000g)_009.fcs	-18.0	24.8	15.9
Specimen_001_008 TP2 1 ml serum+ 900 UL PBS (10000g)_008.fcs	-14.1	24.2	17.3
Specimen_001_008 TP1 1 ml serum+ 900 UL PBS (10000g)_007.fcs	-20.5	22.4	15.4
Specimen_001_008 (200 ul x5)+ 900 ul PBS (IgG)_012.fcs	-16.7	22.6	15.9

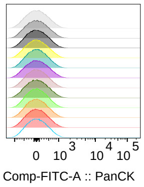

Sample Name	Median : Comp-FITC-A	Mean : Comp-FITC-A	Geometric Mean : Comp-FITC-A
Specimen_001_066 TP5 1 ml serum+ 900 UL PBS (10000g)_006.fcs	25.7	37.9	33.0
Specimen_001_066 TP4 1 ml serum+ 900 UL PBS (10000g)_005.fcs	23.1	35.0	29.4
Specimen_001_066 TP3 1 ml serum+ 900 UL PBS (10000g)_004.fcs	21.8	35.3	30.7
Specimen_001_066 TP2 1 ml serum+ 900 UL PBS (10000g)_003.fcs	23.1	37.5	32.6
Specimen_001_066 TP1 1 ml serum+ 900 UL PBS (10000g)_002.fcs	25.7	36.6	31.6
Specimen_001_066 (200ul x5)+ 900 UL PBS (IgG)_001.fcs	27.0	38.9	33.8
Specimen_001_008 TP5 1 ml serum+ 900 UL PBS (10000g)_011.fcs	24.4	37.0	32.3
Specimen_001_008 TP4 1 ml serum+ 900 UL PBS (10000g)_010.fcs	23.1	33.6	29.0
Specimen_001_008 TP3 1 ml serum+ 900 UL PBS (10000g)_009.fcs	21.8	34.3	29.7
Specimen_001_008 TP2 1 ml serum+ 900 UL PBS (10000g)_008.fcs	21.8	33.7	29.5
Specimen_001_008 TP1 1 ml serum+ 900 UL PBS (10000g)_007.fcs	25.7	38.3	33.4
Specimen_001_008 (200 ul x5)+ 900 ul PBS (IgG)_012.fcs	25.7	38.5	34.0
