# Supplementary material for: Extracellular vesicles as prognostic biomarkers: results of a neoadjuvant chemoimmunotherapy clinical trial in stage IIIA (N2) non-small-cell lung cancer (SAKK 16/14)
Source: Front Immunol. 2026 Jul 1;17:1807542. doi: 10.3389/fimmu.2026.1807542 (PMC13369264; doi:10.3389/fimmu.2026.1807542)
Supplement: Supplementary Figure 1 — Trial design and extracellular vesicle isolation workflow. Trial design adapted from Rothschild, Sacha I., et al. “SAKK 16/14: durvalumab in addition to neoadjuvant chemotherapy in patients with stage IIIA (N2) non–small-cell lung cancer—a multicenter single-arm phase II trial.” (a) Workflow of extracellular vesicle (EV) isolation and characterization adapted from Benecke, Laura et al. “Isolation and analysis of tumor−derived extracellular vesicles from head and neck squamous cell carcinoma plasma by galectin−based glycan recognition particles.” Created in BioRender. Chiang, M. (2025) https://BioRender.com/7sfvuh0 (b). [file DataSheet1.zip › Gated_Raw_flow_data/(010 + 016) MFI.pdf]

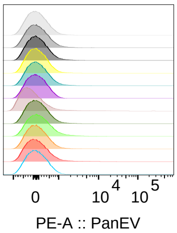

Sample Name	Median : PE-A	Mean : PE-A	Geometric Mean : PE-A
Specimen_001_016_TP5_1 ml_EV staining_012.fcs	17.9	127	45.0
Specimen_001_016_TP4_1 ml_EV staining_011.fcs	34.4	268	78.0
Specimen_001_016_TP3_1 ml_EV staining_010.fcs	35.9	135	71.9
Specimen_001_016_TP2_1 ml_EV staining_009.fcs	22.4	180	52.9
Specimen_001_016_TP1_1 ml_EV staining_008.fcs	38.9	205	77.6
Specimen_001_016_TP1-5_total_1 ml_IgG_007.fcs	13.5	41.9	26.6
Specimen_001_010_TP5_1 ml_EV staining_006.fcs	-184	693	-59.4
Specimen_001_010_TP4_1 ml_EV staining_005.fcs	34.4	143	70.9
Specimen_001_010_TP3_1 ml_EV staining_004.fcs	96.0	292	155
Specimen_001_010_TP2_1 ml_EV staining_003.fcs	26.9	212	68.8
Specimen_001_010_TP1_1 ml_EV staining_002.fcs	17.9	339	84.3
Specimen_001_010_TP1-5_total_1 ml_IgG_001.fcs	10.5	54.3	28.9

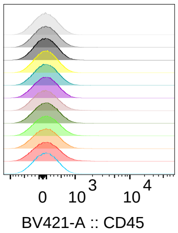

Sample Name	Median : BV421-A	Mean : BV421-A	Geometric Mean : BV421-A
Specimen_001_016_TP5_1 ml_EV staining_012.fcs	85.2	97.4	86.8
Specimen_001_016_TP4_1 ml_EV staining_011.fcs	94.0	132	103
Specimen_001_016_TP3_1 ml_EV staining_010.fcs	88.5	103	92.0
Specimen_001_016_TP2_1 ml_EV staining_009.fcs	92.9	103	94.9
Specimen_001_016_TP1_1 ml_EV staining_008.fcs	99.4	124	106
Specimen_001_016_TP1-5_total_1 ml_IgG_007.fcs	91.8	95.4	90.0
Specimen_001_010_TP5_1 ml_EV staining_006.fcs	86.3	172	110
Specimen_001_010_TP4_1 ml_EV staining_005.fcs	103	120	108
Specimen_001_010_TP3_1 ml_EV staining_004.fcs	112	137	119
Specimen_001_010_TP2_1 ml_EV staining_003.fcs	97.2	116	101
Specimen_001_010_TP1_1 ml_EV staining_002.fcs	107	150	122
Specimen_001_010_TP1-5_total_1 ml_IgG_001.fcs	89.6	104	90.5

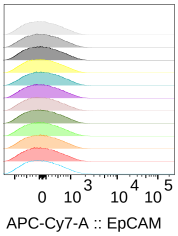

Sample Name	Median : APC-Cy7-A	Mean : APC-Cy7-A	Geometric Mean : APC-Cy7-A
Specimen_001_016_TP5_1 ml_EV staining_012.fcs	-10.3	28.9	13.2
Specimen_001_016_TP4_1 ml_EV staining_011.fcs	6.42	48.2	27.2
Specimen_001_016_TP3_1 ml_EV staining_010.fcs	2.57	41.1	25.3
Specimen_001_016_TP2_1 ml_EV staining_009.fcs	-1.28	30.7	20.0
Specimen_001_016_TP1_1 ml_EV staining_008.fcs	30.8	66.3	49.6
Specimen_001_016_TP1-5_total_1 ml_IgG_007.fcs	-6.42	29.4	17.8
Specimen_001_010_TP5_1 ml_EV staining_006.fcs	-10.3	63.0	17.6
Specimen_001_010_TP4_1 ml_EV staining_005.fcs	-6.42	25.8	15.8
Specimen_001_010_TP3_1 ml_EV staining_004.fcs	-5.13	36.6	19.7
Specimen_001_010_TP2_1 ml_EV staining_003.fcs	-1.07E-13	40.8	23.9
Specimen_001_010_TP1_1 ml_EV staining_002.fcs	-7.70	39.0	18.4
Specimen_001_010_TP1-5_total_1 ml_IgG_001.fcs	-18.0	43.0	11.5

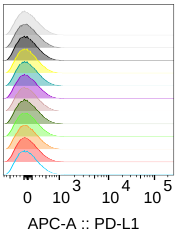

Sample Name	Median : APC-A	Mean : APC-A	Geometric Mean : APC-A
Specimen_001_016_TP5_1 ml_EV staining_012.fcs	-9.64	18.6	14.0
Specimen_001_016_TP4_1 ml_EV staining_011.fcs	-8.57	19.6	15.0
Specimen_001_016_TP3_1 ml_EV staining_010.fcs	-9.64	16.4	12.6
Specimen_001_016_TP2_1 ml_EV staining_009.fcs	-10.7	15.1	11.7
Specimen_001_016_TP1_1 ml_EV staining_008.fcs	-6.43	20.8	17.0
Specimen_001_016_TP1-5_total_1 ml_IgG_007.fcs	-8.57	17.4	13.6
Specimen_001_010_TP5_1 ml_EV staining_006.fcs	-8.57	18.2	13.8
Specimen_001_010_TP4_1 ml_EV staining_005.fcs	-10.7	16.5	13.0
Specimen_001_010_TP3_1 ml_EV staining_004.fcs	-8.57	18.6	14.6
Specimen_001_010_TP2_1 ml_EV staining_003.fcs	-8.57	19.4	15.5
Specimen_001_010_TP1_1 ml_EV staining_002.fcs	-8.57	21.1	16.3
Specimen_001_010_TP1-5_total_1 ml_IgG_001.fcs	-8.57	24.5	14.9

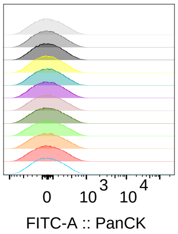

Sample Name	Median : FITC-A	Mean : FITC-A	Geometric Mean : FITC-A
Specimen_001_016_TP5_1 ml_EV staining_012.fcs	10.7	20.7	16.8
Specimen_001_016_TP4_1 ml_EV staining_011.fcs	7.66	22.1	13.9
Specimen_001_016_TP3_1 ml_EV staining_010.fcs	9.95	20.0	14.4
Specimen_001_016_TP2_1 ml_EV staining_009.fcs	7.66	16.6	13.7
Specimen_001_016_TP1_1 ml_EV staining_008.fcs	12.3	21.0	17.2
Specimen_001_016_TP1-5_total_1 ml_IgG_007.fcs	11.5	20.6	16.0
Specimen_001_010_TP5_1 ml_EV staining_006.fcs	11.5	30.3	17.6
Specimen_001_010_TP4_1 ml_EV staining_005.fcs	11.5	19.8	16.3
Specimen_001_010_TP3_1 ml_EV staining_004.fcs	17.6	27.6	21.7
Specimen_001_010_TP2_1 ml_EV staining_003.fcs	12.3	20.1	16.4
Specimen_001_010_TP1_1 ml_EV staining_002.fcs	13.0	24.1	17.7
Specimen_001_010_TP1-5_total_1 ml_IgG_001.fcs	9.19	16.9	13.5
