# Supplementary material for: Extracellular vesicles as prognostic biomarkers: results of a neoadjuvant chemoimmunotherapy clinical trial in stage IIIA (N2) non-small-cell lung cancer (SAKK 16/14)
Source: Front Immunol. 2026 Jul 1;17:1807542. doi: 10.3389/fimmu.2026.1807542 (PMC13369264; doi:10.3389/fimmu.2026.1807542)
Supplement: Supplementary Figure 1 — Trial design and extracellular vesicle isolation workflow. Trial design adapted from Rothschild, Sacha I., et al. “SAKK 16/14: durvalumab in addition to neoadjuvant chemotherapy in patients with stage IIIA (N2) non–small-cell lung cancer—a multicenter single-arm phase II trial.” (a) Workflow of extracellular vesicle (EV) isolation and characterization adapted from Benecke, Laura et al. “Isolation and analysis of tumor−derived extracellular vesicles from head and neck squamous cell carcinoma plasma by galectin−based glycan recognition particles.” Created in BioRender. Chiang, M. (2025) https://BioRender.com/7sfvuh0 (b). [file DataSheet1.zip › Gated_Raw_flow_data/(018) MFI.pdf]

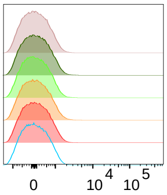

Comp-PE-A :: PanEV

	Sample Name	Median : Comp-PE-A	Mean : Comp-PE-A	Geometric Mean : Comp-PE-A
	Specimen_001_018 T5 (1 ml 10000g)+ 900 ul PBS_012.fcs	38.9	71.1	51.5
	Specimen_001_018 T4 (1 ml 10000g)+ 900 ul PBS_011.fcs	58.4	101	73.0
	Specimen_001_018 T3 (1 ml 10000g)+ 900 ul PBS_010.fcs	35.9	78.4	54.0
	Specimen_001_018 T2 (1 ml 10000g)+ 900 ul PBS_009.fcs	44.9	126	61.9
	Specimen_001_018 T1 (1 ml 10000g)+ 900 ul PBS_008.fcs	52.4	132	67.0
	Specimen_001_018 (200ul x5)+ 900 UL PBS (IgG)_007.fcs	23.9	60.9	38.3

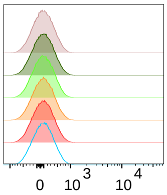

Comp-BV421-A :: CD45

	Sample Name	Median : Comp-BV421-A	Mean : Comp-BV421-A	Geometric Mean : Comp-BV42
	Specimen_001_018 T5 (1 ml 10000g)+ 900 ul PBS_012.fcs	92.9	95.6	
	Specimen_001_018 T4 (1 ml 10000g)+ 900 ul PBS_011.fcs	94.0	96.0	
	Specimen_001_018 T3 (1 ml 10000g)+ 900 ul PBS_010.fcs	94.0	98.4	
	Specimen_001_018 T2 (1 ml 10000g)+ 900 ul PBS_009.fcs	94.0	103	
	Specimen_001_018 T1 (1 ml 10000g)+ 900 ul PBS_008.fcs	92.9	101	
	Specimen_001_018 (200ul x5)+ 900 UL PBS (IgG)_007.fcs	92.9	95.4	

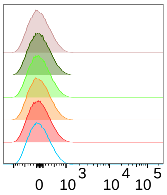

Comp-APC-Cy7-A :: EpCAM

	Sample Name	Median : Comp-APC-Cy7-A	Mean : Comp-APC-Cy7-A	Geometric Mean : Comp-
	Specimen_001_018 T5 (1 ml 10000g)+ 900 ul PBS_012.fcs	-18.0	1.25	
	Specimen_001_018 T4 (1 ml 10000g)+ 900 ul PBS_011.fcs	-3.85	14.1	
	Specimen_001_018 T3 (1 ml 10000g)+ 900 ul PBS_010.fcs	-10.3	13.0	
	Specimen_001_018 T2 (1 ml 10000g)+ 900 ul PBS_009.fcs	-11.6	24.3	
	Specimen_001_018 T1 (1 ml 10000g)+ 900 ul PBS_008.fcs	-16.7	22.2	
	Specimen_001_018 (200ul x5)+ 900 UL PBS (IgG)_007.fcs	-14.1	7.44	

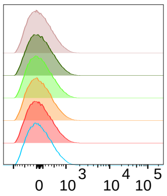

Comp-APC-A :: PD-L1

	Sample Name	Median : Comp-APC-A	Mean : Comp-APC-A	Geometric Mean : Con
	Specimen_001_018 T5 (1 ml 10000g)+ 900 ul PBS_012.fcs	-10.3	29.3	
	Specimen_001_018 T4 (1 ml 10000g)+ 900 ul PBS_011.fcs	7.70	49.5	
	Specimen_001_018 T3 (1 ml 10000g)+ 900 ul PBS_010.fcs	-5.13	37.8	
	Specimen_001_018 T2 (1 ml 10000g)+ 900 ul PBS_009.fcs	-12.8	35.2	
	Specimen_001_018 T1 (1 ml 10000g)+ 900 ul PBS_008.fcs	-11.6	35.3	
	Specimen_001_018 (200ul x5)+ 900 UL PBS (IgG)_007.fcs	-11.6	52.7	

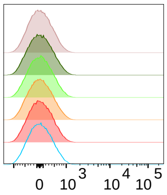

Comp-FITC-A :: PanCK

	Sample Name	Median : Comp-FITC-A	Mean : Comp-FITC-A	Geometric Mean : Comp-
	Specimen_001_018 T5 (1 ml 10000g)+ 900 ul PBS_012.fcs	19.3	32.3	
	Specimen_001_018 T4 (1 ml 10000g)+ 900 ul PBS_011.fcs	20.5	33.5	
	Specimen_001_018 T3 (1 ml 10000g)+ 900 ul PBS_010.fcs	24.4	33.6	
	Specimen_001_018 T2 (1 ml 10000g)+ 900 ul PBS_009.fcs	21.8	35.0	
	Specimen_001_018 T1 (1 ml 10000g)+ 900 ul PBS_008.fcs	24.4	38.5	
	Specimen_001_018 (200ul x5)+ 900 UL PBS (IgG)_007.fcs	20.5	34.4	

1-A
91.1
90.7
91.4
91.3
90.9
90.6

-APC-Cy7-A
-0.78
11.1
6.06
5.54
0.84
3.68

np-APC-A
22.2
40.3
27.6
21.2
22.6
23.0

-FITC-A
28.7
29.4
29.6
30.6
34.2
29.8
