# Supplementary material for: Extracellular vesicles as prognostic biomarkers: results of a neoadjuvant chemoimmunotherapy clinical trial in stage IIIA (N2) non-small-cell lung cancer (SAKK 16/14)
Source: Front Immunol. 2026 Jul 1;17:1807542. doi: 10.3389/fimmu.2026.1807542 (PMC13369264; doi:10.3389/fimmu.2026.1807542)
Supplement: Supplementary Figure 1 — Trial design and extracellular vesicle isolation workflow. Trial design adapted from Rothschild, Sacha I., et al. “SAKK 16/14: durvalumab in addition to neoadjuvant chemotherapy in patients with stage IIIA (N2) non–small-cell lung cancer—a multicenter single-arm phase II trial.” (a) Workflow of extracellular vesicle (EV) isolation and characterization adapted from Benecke, Laura et al. “Isolation and analysis of tumor−derived extracellular vesicles from head and neck squamous cell carcinoma plasma by galectin−based glycan recognition particles.” Created in BioRender. Chiang, M. (2025) https://BioRender.com/7sfvuh0 (b). [file DataSheet1.zip › Gated_Raw_flow_data/(053+055) MFI.pdf]

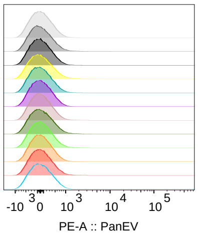

Sample Name	Median : PE-A	Mean : PE-A	Geometric Mean : PE-A
Specimen_001_055_TP5_1 ml_EV staining_012.fcs	15.0	55.8	32.8
Specimen_001_055_TP4_1 ml_EV staining_011.fcs	4.49	235	48.1
Specimen_001_055_TP3_1 ml_EV staining_010.fcs	2.99	123	27.4
Specimen_001_055_TP2_1 ml_EV staining_009.fcs	0	266	50.7
Specimen_001_055_TP1_1 ml_EV staining_008.fcs	1.50	138	28.3
Specimen_001_055_TP1-5_total_1 ml_IgG_007.fcs	12.0	31.1	25.7
Specimen_001_053_TP5_1 ml_EV staining_006.fcs	15.0	138	42.0
Specimen_001_053_TP4_1 ml_EV staining_005.fcs	19.4	178	51.9
Specimen_001_053_TP3_1 ml_EV staining_004.fcs	19.4	125	48.0
Specimen_001_053_TP2_1 ml_EV staining_003.fcs	19.4	83.7	39.5
Specimen_001_053_TP1_1 ml_EV staining_002.fcs	15.0	133	41.8
Specimen_001_053_TP1-5_total_1 ml_IgG_001.fcs	8.97	28.3	22.9

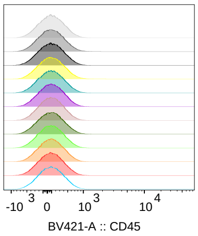

Sample Name	Median : BV421-A	Mean : BV421-A	Geometric Mean : BV421-A
Specimen_001_055_TP5_1 ml_EV staining_012.fcs	104	112	104
Specimen_001_055_TP4_1 ml_EV staining_011.fcs	102	130	109
Specimen_001_055_TP3_1 ml_EV staining_010.fcs	98.3	111	99.7
Specimen_001_055_TP2_1 ml_EV staining_009.fcs	102	140	110
Specimen_001_055_TP1_1 ml_EV staining_008.fcs	99.4	110	100.0
Specimen_001_055_TP1-5_total_1 ml_IgG_007.fcs	102	106	100.0
Specimen_001_053_TP5_1 ml_EV staining_006.fcs	101	109	100
Specimen_001_053_TP4_1 ml_EV staining_005.fcs	95.1	109	96.3
Specimen_001_053_TP3_1 ml_EV staining_004.fcs	95.1	103	95.3
Specimen_001_053_TP2_1 ml_EV staining_003.fcs	97.2	105	97.5
Specimen_001_053_TP1_1 ml_EV staining_002.fcs	95.1	107	96.3
Specimen_001_053_TP1-5_total_1 ml_IgG_001.fcs	97.2	104	97.7

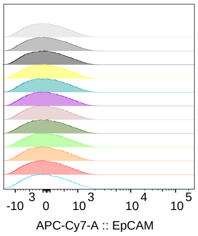

Sample Name	Median : APC-Cy7-A	Mean : APC-Cy7-A	Geometric Mean : APC-Cy7-A
Specimen_001_055_TP5_1 ml_EV staining_012.fcs	-14.1	21.2	9.84
Specimen_001_055_TP4_1 ml_EV staining_011.fcs	-6.42	36.6	18.6
Specimen_001_055_TP3_1 ml_EV staining_010.fcs	-19.3	21.5	5.71
Specimen_001_055_TP2_1 ml_EV staining_009.fcs	-18.0	33.3	8.77
Specimen_001_055_TP1_1 ml_EV staining_008.fcs	-11.6	28.7	11.3
Specimen_001_055_TP1-5_total_1 ml_IgG_007.fcs	-14.1	21.5	9.30
Specimen_001_053_TP5_1 ml_EV staining_006.fcs	-15.4	20.9	10.3
Specimen_001_053_TP4_1 ml_EV staining_005.fcs	-2.57	34.3	20.7
Specimen_001_053_TP3_1 ml_EV staining_004.fcs	12.8	49.7	35.6
Specimen_001_053_TP2_1 ml_EV staining_003.fcs	-18.0	19.9	7.36
Specimen_001_053_TP1_1 ml_EV staining_002.fcs	-21.8	19.1	4.90
Specimen_001_053_TP1-5_total_1 ml_IgG_001.fcs	-16.7	20.9	8.29

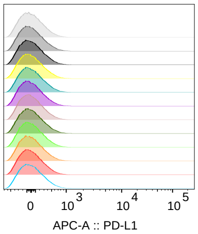

Sample Name	Median : APC-A	Mean : APC-A	Geometric Mean : APC-A
Specimen_001_055_TP5_1 ml_EV staining_012.fcs	-6.43	18.8	15.1
Specimen_001_055_TP4_1 ml_EV staining_011.fcs	-9.64	17.6	13.9
Specimen_001_055_TP3_1 ml_EV staining_010.fcs	-8.57	17.8	13.5
Specimen_001_055_TP2_1 ml_EV staining_009.fcs	-11.8	15.4	11.4
Specimen_001_055_TP1_1 ml_EV staining_008.fcs	-9.64	19.2	15.3
Specimen_001_055_TP1-5_total_1 ml_IgG_007.fcs	-13.9	16.9	9.72
Specimen_001_053_TP5_1 ml_EV staining_006.fcs	-9.64	16.7	13.3
Specimen_001_053_TP4_1 ml_EV staining_005.fcs	-6.43	19.1	15.4
Specimen_001_053_TP3_1 ml_EV staining_004.fcs	-7.50	19.8	15.8
Specimen_001_053_TP2_1 ml_EV staining_003.fcs	-9.64	16.9	13.2
Specimen_001_053_TP1_1 ml_EV staining_002.fcs	-10.7	15.0	11.5
Specimen_001_053_TP1-5_total_1 ml_IgG_001.fcs	-5.36	21.8	17.0

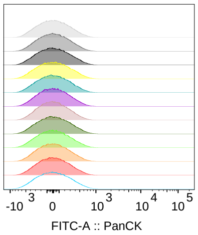

Sample Name	Median : FITC-A	Mean : FITC-A	Geometric Mean : FITC-A
Specimen_001_055_TP5_1 ml_EV staining_012.fcs	12.3	20.1	16.6
Specimen_001_055_TP4_1 ml_EV staining_011.fcs	14.5	32.0	19.6
Specimen_001_055_TP3_1 ml_EV staining_010.fcs	9.95	22.1	15.8
Specimen_001_055_TP2_1 ml_EV staining_009.fcs	13.0	38.1	17.8
Specimen_001_055_TP1_1 ml_EV staining_008.fcs	10.7	21.8	15.9
Specimen_001_055_TP1-5_total_1 ml_IgG_007.fcs	12.3	19.7	16.9
Specimen_001_053_TP5_1 ml_EV staining_006.fcs	6.89	15.4	12.9
Specimen_001_053_TP4_1 ml_EV staining_005.fcs	14.5	24.9	20.8
Specimen_001_053_TP3_1 ml_EV staining_004.fcs	9.95	18.6	15.4
Specimen_001_053_TP2_1 ml_EV staining_003.fcs	9.95	19.6	14.1
Specimen_001_053_TP1_1 ml_EV staining_002.fcs	11.5	20.6	16.6
Specimen_001_053_TP1-5_total_1 ml_IgG_001.fcs	11.5	19.5	16.4
