# Supplementary material for: Extracellular vesicles as prognostic biomarkers: results of a neoadjuvant chemoimmunotherapy clinical trial in stage IIIA (N2) non-small-cell lung cancer (SAKK 16/14)
Source: Front Immunol. 2026 Jul 1;17:1807542. doi: 10.3389/fimmu.2026.1807542 (PMC13369264; doi:10.3389/fimmu.2026.1807542)
Supplement: Supplementary Figure 1 — Trial design and extracellular vesicle isolation workflow. Trial design adapted from Rothschild, Sacha I., et al. “SAKK 16/14: durvalumab in addition to neoadjuvant chemotherapy in patients with stage IIIA (N2) non–small-cell lung cancer—a multicenter single-arm phase II trial.” (a) Workflow of extracellular vesicle (EV) isolation and characterization adapted from Benecke, Laura et al. “Isolation and analysis of tumor−derived extracellular vesicles from head and neck squamous cell carcinoma plasma by galectin−based glycan recognition particles.” Created in BioRender. Chiang, M. (2025) https://BioRender.com/7sfvuh0 (b). [file DataSheet1.zip › Gated_Raw_flow_data/(23 + 39) MFI.pdf]

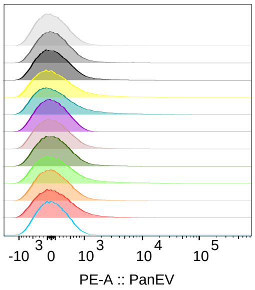


| Sample Name | Median : PE-A | Mean : PE-A | Geometric Mean : PE-A |
|---|---|---|---|
| Specimen_001_039_TP5_1 ml_EV staining_012.fcs | 15.0 | 221 | 59.9 |
| Specimen_001_039_TP4_1 ml_EV staining_011.fcs | 53.9 | 282 | 105 |
| Specimen_001_039_TP3_1 ml_EV staining_010.fcs | 46.4 | 362 | 100 |
| Specimen_001_039_TP2_1 ml_EV staining_009.fcs | 50.9 | 698 | 164 |
| Specimen_001_039_TP1_1 ml_EV staining_008.fcs | 53.9 | 936 | 182 |
| Specimen_001_039_TP1-5_total_1 ml_IgG_007.fcs | 13.5 | 36.2 | 28.6 |
| Specimen_001_023_TP5_1 ml_EV staining_006.fcs | 41.9 | 240 | 91.0 |
| Specimen_001_023_TP4_1 ml_EV staining_005.fcs | 52.4 | 257 | 98.5 |
| Specimen_001_023_TP3_1 ml_EV staining_004.fcs | 79.4 | 678 | 191 |
| Specimen_001_023_TP2_1 ml_EV staining_003.fcs | 34.4 | 232 | 79.1 |
| Specimen_001_023_TP1_1 ml_EV staining_002.fcs | 67.4 | 440 | 133 |
| Specimen_001_023_TP1-5_total_1 ml_IgG_001.fcs | 17.9 | 38.7 | 31.5 |

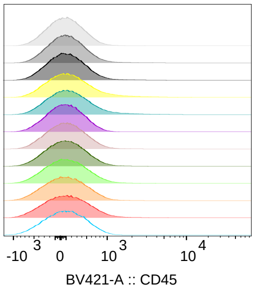


| Sample Name | Median : BV421-A | Mean : BV421-A | Geometric Mean : BV421-A |
|---|---|---|---|
| Specimen_001_039_TP5_1 ml_EV staining_012.fcs | 96.2 | 128 | 105 |
| Specimen_001_039_TP4_1 ml_EV staining_011.fcs | 117 | 179 | 140 |
| Specimen_001_039_TP3_1 ml_EV staining_010.fcs | 113 | 178 | 133 |
| Specimen_001_039_TP2_1 ml_EV staining_009.fcs | 151 | 344 | 209 |
| Specimen_001_039_TP1_1 ml_EV staining_008.fcs | 154 | 293 | 198 |
| Specimen_001_039_TP1-5_total_1 ml_IgG_007.fcs | 95.1 | 105 | 96.1 |
| Specimen_001_023_TP5_1 ml_EV staining_006.fcs | 109 | 143 | 119 |
| Specimen_001_023_TP4_1 ml_EV staining_005.fcs | 107 | 146 | 117 |
| Specimen_001_023_TP3_1 ml_EV staining_004.fcs | 116 | 163 | 130 |
| Specimen_001_023_TP2_1 ml_EV staining_003.fcs | 97.2 | 124 | 105 |
| Specimen_001_023_TP1_1 ml_EV staining_002.fcs | 120 | 192 | 138 |
| Specimen_001_023_TP1-5_total_1 ml_IgG_001.fcs | 98.3 | 103 | 94.6 |

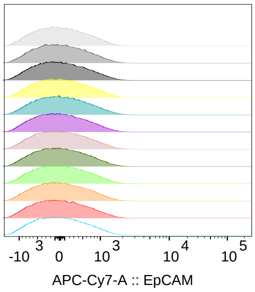


| Sample Name | Median : APC-Cy7-A | Mean : APC-Cy7-A | Geometric Mean : APC-Cy7-A |
|---|---|---|---|
| Specimen_001_039_TP5_1 ml_EV staining_012.fcs | -2.57 | 36.4 | 22.0 |
| Specimen_001_039_TP4_1 ml_EV staining_011.fcs | -1.07E-13 | 41.3 | 23.5 |
| Specimen_001_039_TP3_1 ml_EV staining_010.fcs | -8.98 | 39.0 | 17.2 |
| Specimen_001_039_TP2_1 ml_EV staining_009.fcs | 12.8 | 69.9 | 37.6 |
| Specimen_001_039_TP1_1 ml_EV staining_008.fcs | 18.0 | 79.8 | 44.9 |
| Specimen_001_039_TP1-5_total_1 ml_IgG_007.fcs | -8.98 | 35.8 | 18.0 |
| Specimen_001_023_TP5_1 ml_EV staining_006.fcs | 2.57 | 62.2 | 28.1 |
| Specimen_001_023_TP4_1 ml_EV staining_005.fcs | -1.07E-13 | 36.5 | 22.2 |
| Specimen_001_023_TP3_1 ml_EV staining_004.fcs | 7.70 | 51.4 | 30.0 |
| Specimen_001_023_TP2_1 ml_EV staining_003.fcs | -1.07E-13 | 42.5 | 25.1 |
| Specimen_001_023_TP1_1 ml_EV staining_002.fcs | -7.70 | 39.1 | 18.3 |
| Specimen_001_023_TP1-5_total_1 ml_IgG_001.fcs | -10.3 | 23.9 | 12.9 |

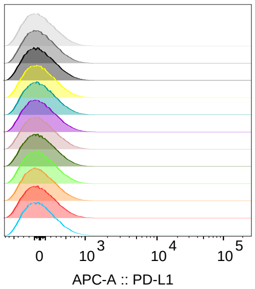


| Sample Name | Median : APC-A | Mean : APC-A | Geometric Mean : APC-A |
|---|---|---|---|
| Specimen_001_039_TP5_1 ml_EV staining_012.fcs | -11.8 | 16.0 | 12.0 |
| Specimen_001_039_TP4_1 ml_EV staining_011.fcs | -4.29 | 26.6 | 19.4 |
| Specimen_001_039_TP3_1 ml_EV staining_010.fcs | -9.64 | 18.9 | 14.4 |
| Specimen_001_039_TP2_1 ml_EV staining_009.fcs | -5.36 | 23.0 | 18.1 |
| Specimen_001_039_TP1_1 ml_EV staining_008.fcs | -1.07 | 29.0 | 23.5 |
| Specimen_001_039_TP1-5_total_1 ml_IgG_007.fcs | -6.43 | 25.2 | 18.7 |
| Specimen_001_023_TP5_1 ml_EV staining_006.fcs | -9.64 | 18.6 | 13.6 |
| Specimen_001_023_TP4_1 ml_EV staining_005.fcs | -6.43 | 20.5 | 16.2 |
| Specimen_001_023_TP3_1 ml_EV staining_004.fcs | -5.36 | 22.0 | 17.5 |
| Specimen_001_023_TP2_1 ml_EV staining_003.fcs | -11.8 | 17.0 | 12.3 |
| Specimen_001_023_TP1_1 ml_EV staining_002.fcs | -9.64 | 18.5 | 14.0 |
| Specimen_001_023_TP1-5_total_1 ml_IgG_001.fcs | -7.50 | 20.6 | 15.9 |

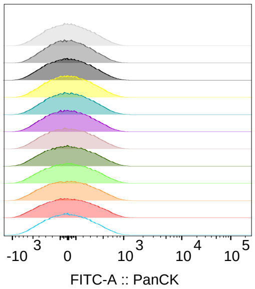


| Sample Name | Median : FITC-A | Mean : FITC-A | Geometric Mean : FITC-A |
|---|---|---|---|
| Specimen_001_039_TP5_1 ml_EV staining_012.fcs | 13.0 | 27.5 | 18.5 |
| Specimen_001_039_TP4_1 ml_EV staining_011.fcs | 11.5 | 25.5 | 16.7 |
| Specimen_001_039_TP3_1 ml_EV staining_010.fcs | 12.3 | 28.5 | 16.4 |
| Specimen_001_039_TP2_1 ml_EV staining_009.fcs | 19.1 | 37.8 | 24.2 |
| Specimen_001_039_TP1_1 ml_EV staining_008.fcs | 18.4 | 31.2 | 23.5 |
| Specimen_001_039_TP1-5_total_1 ml_IgG_007.fcs | 12.3 | 20.3 | 16.8 |
| Specimen_001_023_TP5_1 ml_EV staining_006.fcs | 13.0 | 32.2 | 17.8 |
| Specimen_001_023_TP4_1 ml_EV staining_005.fcs | 11.5 | 23.8 | 16.6 |
| Specimen_001_023_TP3_1 ml_EV staining_004.fcs | 17.6 | 30.1 | 21.4 |
| Specimen_001_023_TP2_1 ml_EV staining_003.fcs | 16.1 | 26.9 | 18.1 |
| Specimen_001_023_TP1_1 ml_EV staining_002.fcs | 15.3 | 33.3 | 19.3 |
| Specimen_001_023_TP1-5_total_1 ml_IgG_001.fcs | 10.7 | 20.2 | 16.5 |
