# Supplementary material for: Extracellular vesicles as prognostic biomarkers: results of a neoadjuvant chemoimmunotherapy clinical trial in stage IIIA (N2) non-small-cell lung cancer (SAKK 16/14)
Source: Front Immunol. 2026 Jul 1;17:1807542. doi: 10.3389/fimmu.2026.1807542 (PMC13369264; doi:10.3389/fimmu.2026.1807542)
Supplement: Supplementary Figure 1 — Trial design and extracellular vesicle isolation workflow. Trial design adapted from Rothschild, Sacha I., et al. “SAKK 16/14: durvalumab in addition to neoadjuvant chemotherapy in patients with stage IIIA (N2) non–small-cell lung cancer—a multicenter single-arm phase II trial.” (a) Workflow of extracellular vesicle (EV) isolation and characterization adapted from Benecke, Laura et al. “Isolation and analysis of tumor−derived extracellular vesicles from head and neck squamous cell carcinoma plasma by galectin−based glycan recognition particles.” Created in BioRender. Chiang, M. (2025) https://BioRender.com/7sfvuh0 (b). [file DataSheet1.zip › Gated_Raw_flow_data/(41 + 60) MFI.pdf]

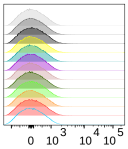

Comp-PE-A :: PanEV

Sample Name	Median : Comp-PE-A	Mean : Comp-PE-A	Geometric Mean : Comp-PE-A
Specimen_001_060 T5 (1 ml 10000g)+ 900 ul PBS_012.fcs	52.6	99.9	73.8
Specimen_001_060 T4 (1 ml 10000g)+ 900 ul PBS_011.fcs	56.5	207	82.4
Specimen_001_060 T3 (1 ml 10000g)+ 900 ul PBS_010.fcs	79.6	229	106
Specimen_001_060 T2 (1 ml 10000g)+ 900 ul PBS_009.fcs	61.6	397	98.7
Specimen_001_060 T1 (1 ml 10000g)+ 900 ul PBS_008.fcs	51.4	97.8	71.9
Specimen_001_060 (200ul x5)+ 900 UL PBS (IgG)_007.fcs	39.8	70.7	53.4
Specimen_001_041 T5 (700 ul 10000g)+ 1200 ul PBS_006.fcs	46.2	86.3	65.4
Specimen_001_041 T4 (700 ul 10000g)+ 1200 ul PBS_005.fcs	39.8	80.4	59.9
Specimen_001_041 T3 (700 ul 10000g)+ 1200 ul PBS_004.fcs	52.6	96.0	71.0
Specimen_001_041 T2 (700 ul 10000g)+ 1200 ul PBS_003.fcs	42.4	98.3	64.2
Specimen_001_041 T1 (700 ul 10000g)+ 1200 ul PBS_002.fcs	46.2	91.2	68.0
Specimen_001_041 (700 ul)+ 1200 UL PBS (IgG)_001.fcs	19.3	46.4	36.2

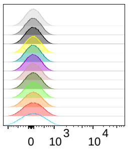

Comp-BV421-A :: CD45

Sample Name	Median : Comp-BV421-A	Mean : Comp-BV421-A	Geometric Mean : Comp-BV421-A
Specimen_001_060 T5 (1 ml 10000g)+ 900 ul PBS_012.fcs	89.6	91.5	87.3
Specimen_001_060 T4 (1 ml 10000g)+ 900 ul PBS_011.fcs	87.4	107	87.1
Specimen_001_060 T3 (1 ml 10000g)+ 900 ul PBS_010.fcs	87.4	108	88.3
Specimen_001_060 T2 (1 ml 10000g)+ 900 ul PBS_009.fcs	87.4	138	90.3
Specimen_001_060 T1 (1 ml 10000g)+ 900 ul PBS_008.fcs	85.2	89.7	83.6
Specimen_001_060 (200ul x5)+ 900 UL PBS (IgG)_007.fcs	85.2	88.0	83.7
Specimen_001_041 T5 (700 ul 10000g)+ 1200 ul PBS_006.fcs	86.3	89.0	84.6
Specimen_001_041 T4 (700 ul 10000g)+ 1200 ul PBS_005.fcs	86.3	90.8	84.6
Specimen_001_041 T3 (700 ul 10000g)+ 1200 ul PBS_004.fcs	87.4	91.9	86.2
Specimen_001_041 T2 (700 ul 10000g)+ 1200 ul PBS_003.fcs	86.3	88.9	83.2
Specimen_001_041 T1 (700 ul 10000g)+ 1200 ul PBS_002.fcs	86.3	90.9	83.8
Specimen_001_041 (700 ul)+ 1200 UL PBS (IgG)_001.fcs	84.1	86.1	78.7

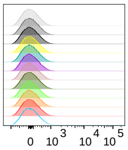

Comp-APC-Cy7-A :: EpCAM

Sample Name	Median : Comp-APC-Cy7-A	Mean : Comp-APC-Cy7-A	Geometric Mean : Comp-APC-Cy7-A
Specimen_001_060 T5 (1 ml 10000g)+ 900 ul PBS_012.fcs	-10.3	8.77	5.72
Specimen_001_060 T4 (1 ml 10000g)+ 900 ul PBS_011.fcs	-10.3	70.5	9.68
Specimen_001_060 T3 (1 ml 10000g)+ 900 ul PBS_010.fcs	-12.8	63.6	6.02
Specimen_001_060 T2 (1 ml 10000g)+ 900 ul PBS_009.fcs	-7.70	160	14.8
Specimen_001_060 T1 (1 ml 10000g)+ 900 ul PBS_008.fcs	-14.1	7.15	1.70
Specimen_001_060 (200ul x5)+ 900 UL PBS (IgG)_007.fcs	-16.7	6.35	1.47
Specimen_001_041 T5 (700 ul 10000g)+ 1200 ul PBS_006.fcs	-12.8	8.57	3.35
Specimen_001_041 T4 (700 ul 10000g)+ 1200 ul PBS_005.fcs	-10.3	13.3	6.34
Specimen_001_041 T3 (700 ul 10000g)+ 1200 ul PBS_004.fcs	-12.8	9.89	5.29
Specimen_001_041 T2 (700 ul 10000g)+ 1200 ul PBS_003.fcs	-12.8	12.6	5.42
Specimen_001_041 T1 (700 ul 10000g)+ 1200 ul PBS_002.fcs	-11.6	9.06	5.40
Specimen_001_041 (700 ul)+ 1200 UL PBS (IgG)_001.fcs	-14.1	3.47	1.38

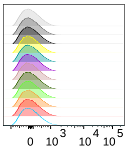

Comp-APC-A :: PD-L1

Sample Name	Median : Comp-APC-A	Mean : Comp-APC-A	Geometric Mean : Comp-APC-A
Specimen_001_060 T5 (1 ml 10000g)+ 900 ul PBS_012.fcs	-14.1	26.9	19.9
Specimen_001_060 T4 (1 ml 10000g)+ 900 ul PBS_011.fcs	-15.4	46.1	19.6
Specimen_001_060 T3 (1 ml 10000g)+ 900 ul PBS_010.fcs	-12.8	49.3	22.6
Specimen_001_060 T2 (1 ml 10000g)+ 900 ul PBS_009.fcs	-14.1	118	23.4
Specimen_001_060 T1 (1 ml 10000g)+ 900 ul PBS_008.fcs	-15.4	25.7	17.7
Specimen_001_060 (200ul x5)+ 900 UL PBS (IgG)_007.fcs	-12.8	61.5	20.9
Specimen_001_041 T5 (700 ul 10000g)+ 1200 ul PBS_006.fcs	-14.1	25.1	17.9
Specimen_001_041 T4 (700 ul 10000g)+ 1200 ul PBS_005.fcs	-15.4	25.4	17.2
Specimen_001_041 T3 (700 ul 10000g)+ 1200 ul PBS_004.fcs	-18.0	22.9	15.2
Specimen_001_041 T2 (700 ul 10000g)+ 1200 ul PBS_003.fcs	-16.7	26.5	17.9
Specimen_001_041 T1 (700 ul 10000g)+ 1200 ul PBS_002.fcs	-14.1	27.2	19.8
Specimen_001_041 (700 ul)+ 1200 UL PBS (IgG)_001.fcs	-16.7	23.2	16.1

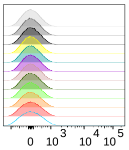

Comp-FITC-A :: PanCK

Sample Name	Median : Comp-FITC-A	Mean : Comp-FITC-A	Geometric Mean : Comp-FITC-A
Specimen_001_060 T5 (1 ml 10000g)+ 900 ul PBS_012.fcs	21.8	34.0	30.5
Specimen_001_060 T4 (1 ml 10000g)+ 900 ul PBS_011.fcs	24.4	41.2	34.8
Specimen_001_060 T3 (1 ml 10000g)+ 900 ul PBS_010.fcs	30.8	46.8	40.7
Specimen_001_060 T2 (1 ml 10000g)+ 900 ul PBS_009.fcs	24.4	47.8	34.7
Specimen_001_060 T1 (1 ml 10000g)+ 900 ul PBS_008.fcs	24.4	37.9	33.1
Specimen_001_060 (200ul x5)+ 900 UL PBS (IgG)_007.fcs	23.1	39.8	33.8
Specimen_001_041 T5 (700 ul 10000g)+ 1200 ul PBS_006.fcs	25.7	38.0	33.9
Specimen_001_041 T4 (700 ul 10000g)+ 1200 ul PBS_005.fcs	23.1	38.6	32.0
Specimen_001_041 T3 (700 ul 10000g)+ 1200 ul PBS_004.fcs	21.8	37.3	31.6
Specimen_001_041 T2 (700 ul 10000g)+ 1200 ul PBS_003.fcs	23.1	37.5	32.9
Specimen_001_041 T1 (700 ul 10000g)+ 1200 ul PBS_002.fcs	21.8	35.3	30.0
Specimen_001_041 (700 ul)+ 1200 UL PBS (IgG)_001.fcs	23.1	35.3	30.4
