# Supplementary material for: Extracellular vesicles as prognostic biomarkers: results of a neoadjuvant chemoimmunotherapy clinical trial in stage IIIA (N2) non-small-cell lung cancer (SAKK 16/14)
Source: Front Immunol. 2026 Jul 1;17:1807542. doi: 10.3389/fimmu.2026.1807542 (PMC13369264; doi:10.3389/fimmu.2026.1807542)
Supplement: Supplementary Figure 1 — Trial design and extracellular vesicle isolation workflow. Trial design adapted from Rothschild, Sacha I., et al. “SAKK 16/14: durvalumab in addition to neoadjuvant chemotherapy in patients with stage IIIA (N2) non–small-cell lung cancer—a multicenter single-arm phase II trial.” (a) Workflow of extracellular vesicle (EV) isolation and characterization adapted from Benecke, Laura et al. “Isolation and analysis of tumor−derived extracellular vesicles from head and neck squamous cell carcinoma plasma by galectin−based glycan recognition particles.” Created in BioRender. Chiang, M. (2025) https://BioRender.com/7sfvuh0 (b). [file DataSheet1.zip › Gated_Raw_flow_data/(49 + 45) MFI.pdf]

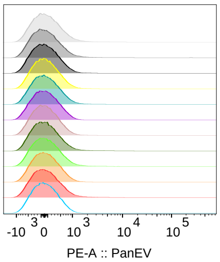

	Sample Name	Median : PE-A	Mean : PE-A	Geometric Mean : PE-A
	Specimen_001_049_TP5_1 ml_EV staining_012.fcs	46.4	387	94.5
	Specimen_001_049_TP4_1 ml_EV staining_011.fcs	16.5	474	68.9
	Specimen_001_049_TP3_1 ml_EV staining_010.fcs	13.5	158	42.2
	Specimen_001_049_TP2_1 ml_EV staining_009.fcs	22.4	189	51.7
	Specimen_001_049_TP1_1 ml_EV staining_008.fcs	22.4	214	54.7
	Specimen_001_049_TP1-5_total_1 ml_IgG_007.fcs	12.0	31.1	24.8
	Specimen_001_045_TP5_1 ml_EV staining_006.fcs	12.0	116	34.9
	Specimen_001_045_TP4_1 ml_EV staining_005.fcs	23.9	264	70.6
	Specimen_001_045_TP3_1 ml_EV staining_004.fcs	34.4	240	74.5
	Specimen_001_045_TP2_1 ml_EV staining_003.fcs	26.9	69.5	46.1
	Specimen_001_045_TP1_1 ml_EV staining_002.fcs	26.9	261	71.3
	Specimen_001_045_TP1-5_total_1 ml_IgG_001.fcs	13.5	31.5	25.8

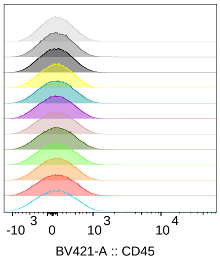

	Sample Name	Median : BV421-A	Mean : BV421-A	Geometric Mean : BV421-A
	Specimen_001_049_TP5_1 ml_EV staining_012.fcs	103	137	111
	Specimen_001_049_TP4_1 ml_EV staining_011.fcs	95.1	132	102
	Specimen_001_049_TP3_1 ml_EV staining_010.fcs	84.1	93.8	84.3
	Specimen_001_049_TP2_1 ml_EV staining_009.fcs	87.4	98.6	86.5
	Specimen_001_049_TP1_1 ml_EV staining_008.fcs	89.6	107	91.1
	Specimen_001_049_TP1-5_total_1 ml_IgG_007.fcs	81.9	86.9	80.0
	Specimen_001_045_TP5_1 ml_EV staining_006.fcs	85.2	98.3	86.0
	Specimen_001_045_TP4_1 ml_EV staining_005.fcs	94.0	112	96.0
	Specimen_001_045_TP3_1 ml_EV staining_004.fcs	92.9	118	96.5
	Specimen_001_045_TP2_1 ml_EV staining_003.fcs	87.4	94.4	86.2
	Specimen_001_045_TP1_1 ml_EV staining_002.fcs	94.0	115	95.2
	Specimen_001_045_TP1-5_total_1 ml_IgG_001.fcs	81.9	87.7	79.6

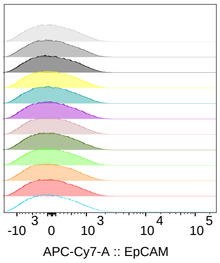

	Sample Name	Median : APC-Cy7-A	Mean : APC-Cy7-A	Geometric Mean : APC-Cy7-A
	Specimen_001_049_TP5_1 ml_EV staining_012.fcs	-6.42	41.1	17.6
	Specimen_001_049_TP4_1 ml_EV staining_011.fcs	-10.3	39.8	16.1
	Specimen_001_049_TP3_1 ml_EV staining_010.fcs	-6.42	36.9	20.3
	Specimen_001_049_TP2_1 ml_EV staining_009.fcs	-8.98	32.6	14.1
	Specimen_001_049_TP1_1 ml_EV staining_008.fcs	-15.4	35.6	12.2
	Specimen_001_049_TP1-5_total_1 ml_IgG_007.fcs	-16.7	21.2	7.84
	Specimen_001_045_TP5_1 ml_EV staining_006.fcs	-8.98	26.5	14.0
	Specimen_001_045_TP4_1 ml_EV staining_005.fcs	-12.8	29.0	11.4
	Specimen_001_045_TP3_1 ml_EV staining_004.fcs	-14.1	31.3	9.82
	Specimen_001_045_TP2_1 ml_EV staining_003.fcs	-23.1	13.2	3.46
	Specimen_001_045_TP1_1 ml_EV staining_002.fcs	-11.6	39.6	14.1
	Specimen_001_045_TP1-5_total_1 ml_IgG_001.fcs	-18.0	22.0	8.81

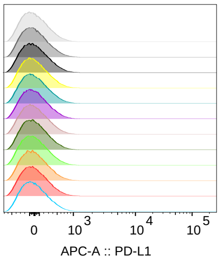

	Sample Name	Median : APC-A	Mean : APC-A	Geometric Mean : APC-A
	Specimen_001_049_TP5_1 ml_EV staining_012.fcs	-11.8	17.8	13.4
	Specimen_001_049_TP4_1 ml_EV staining_011.fcs	-12.9	15.7	11.3
	Specimen_001_049_TP3_1 ml_EV staining_010.fcs	-9.64	17.9	13.9
	Specimen_001_049_TP2_1 ml_EV staining_009.fcs	-11.8	16.9	12.8
	Specimen_001_049_TP1_1 ml_EV staining_008.fcs	-11.8	18.6	13.5
	Specimen_001_049_TP1-5_total_1 ml_IgG_007.fcs	-10.7	16.6	12.5
	Specimen_001_045_TP5_1 ml_EV staining_006.fcs	-11.8	15.0	11.3
	Specimen_001_045_TP4_1 ml_EV staining_005.fcs	-8.57	18.9	14.8
	Specimen_001_045_TP3_1 ml_EV staining_004.fcs	-11.8	15.0	11.0
	Specimen_001_045_TP2_1 ml_EV staining_003.fcs	-9.64	16.2	12.5
	Specimen_001_045_TP1_1 ml_EV staining_002.fcs	-11.8	16.7	12.3
	Specimen_001_045_TP1-5_total_1 ml_IgG_001.fcs	-13.9	20.4	10.7

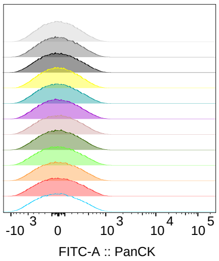

	Sample Name	Median : FITC-A	Mean : FITC-A	Geometric Mean : FITC-A
	Specimen_001_049_TP5_1 ml_EV staining_012.fcs	10.7	35.7	16.0
	Specimen_001_049_TP4_1 ml_EV staining_011.fcs	10.7	29.0	17.0
	Specimen_001_049_TP3_1 ml_EV staining_010.fcs	8.42	22.8	13.7
	Specimen_001_049_TP2_1 ml_EV staining_009.fcs	11.5	32.7	16.2
	Specimen_001_049_TP1_1 ml_EV staining_008.fcs	9.95	30.4	15.4
	Specimen_001_049_TP1-5_total_1 ml_IgG_007.fcs	10.7	19.5	16.0
	Specimen_001_045_TP5_1 ml_EV staining_006.fcs	13.0	24.8	15.9
	Specimen_001_045_TP4_1 ml_EV staining_005.fcs	12.3	25.8	16.9
	Specimen_001_045_TP3_1 ml_EV staining_004.fcs	9.95	32.9	14.8
	Specimen_001_045_TP2_1 ml_EV staining_003.fcs	11.5	22.1	15.9
	Specimen_001_045_TP1_1 ml_EV staining_002.fcs	15.3	35.2	19.4
	Specimen_001_045_TP1-5_total_1 ml_IgG_001.fcs	6.13	13.6	10.3
