# Supplementary material for: Extracellular vesicles as prognostic biomarkers: results of a neoadjuvant chemoimmunotherapy clinical trial in stage IIIA (N2) non-small-cell lung cancer (SAKK 16/14)
Source: Front Immunol. 2026 Jul 1;17:1807542. doi: 10.3389/fimmu.2026.1807542 (PMC13369264; doi:10.3389/fimmu.2026.1807542)
Supplement: Supplementary Figure 1 — Trial design and extracellular vesicle isolation workflow. Trial design adapted from Rothschild, Sacha I., et al. “SAKK 16/14: durvalumab in addition to neoadjuvant chemotherapy in patients with stage IIIA (N2) non–small-cell lung cancer—a multicenter single-arm phase II trial.” (a) Workflow of extracellular vesicle (EV) isolation and characterization adapted from Benecke, Laura et al. “Isolation and analysis of tumor−derived extracellular vesicles from head and neck squamous cell carcinoma plasma by galectin−based glycan recognition particles.” Created in BioRender. Chiang, M. (2025) https://BioRender.com/7sfvuh0 (b). [file DataSheet1.zip › Gated_Raw_flow_data/(49 + 45) PanEV+ PDL1+.pdf]

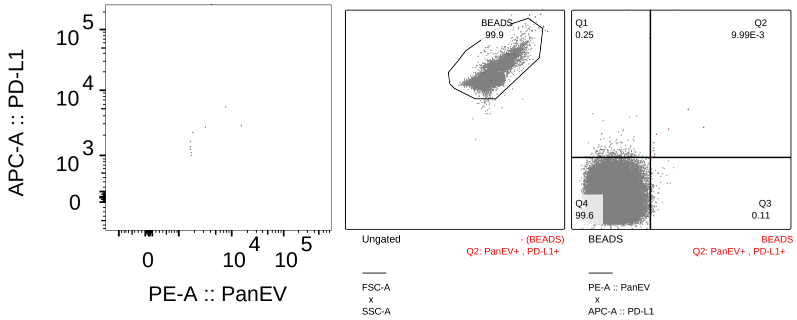

Sample Name	Freq. of BEADS
Specimen_001_045_TP1-5_total_1 ml_IgG_001.fcs	9.99E-3

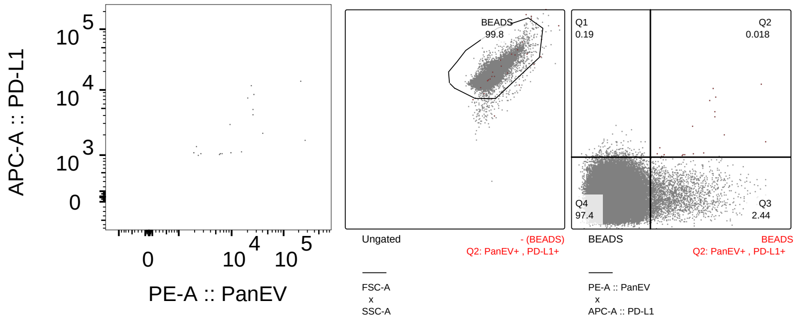

Sample Name	Freq. of BEADS
Specimen_001_045_TP4_1 ml_EV staining_005.fcs	0.018

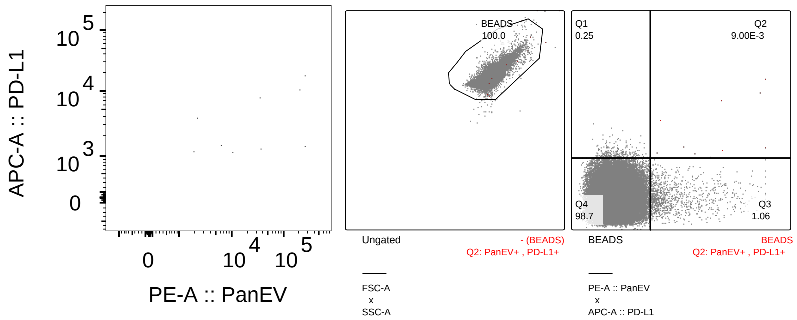

Sample Name	Freq. of BEADS
Specimen_001_049_TP2_1 ml_EV staining_009.fcs	9.00E-3

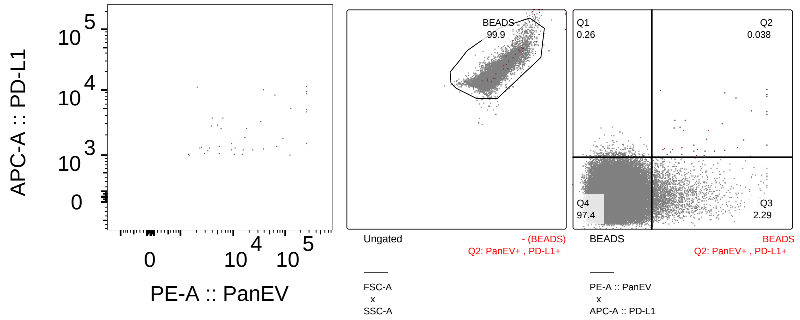

	Sample Name	Freq. of BEADS
▮	Specimen_001_045_TP1_1 ml_EV staining_002.fcs	0.038

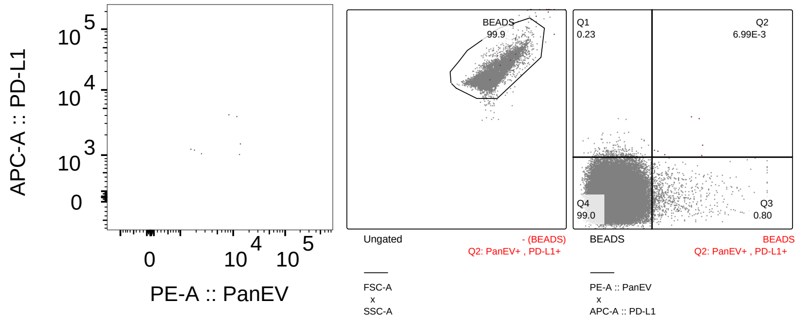

	Sample Name	Freq. of BEADS
▮	Specimen_001_045_TP5_1 ml_EV staining_006.fcs	6.99E-3

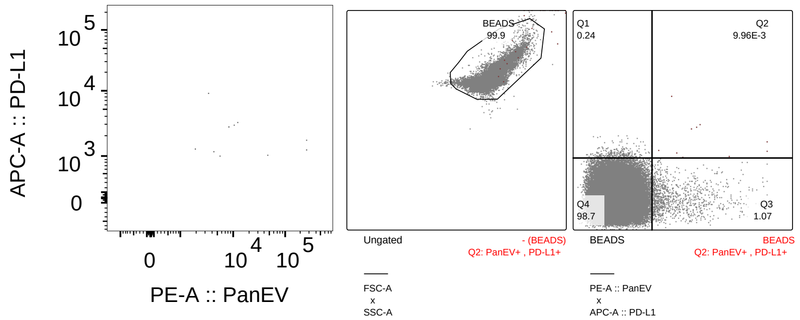

	Sample Name	Freq. of BEADS
▮	Specimen_001_049_TP3_1 ml_EV staining_010.fcs	9.96E-3

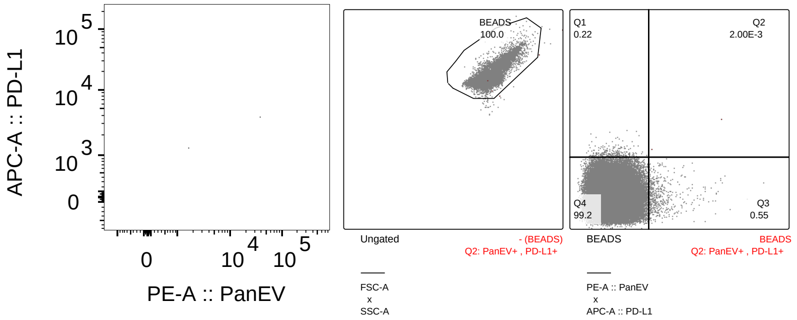

Sample Name	Freq. of BEADS
Specimen_001_045_TP2_1 ml_EV staining_003.fcs	2.00E-3

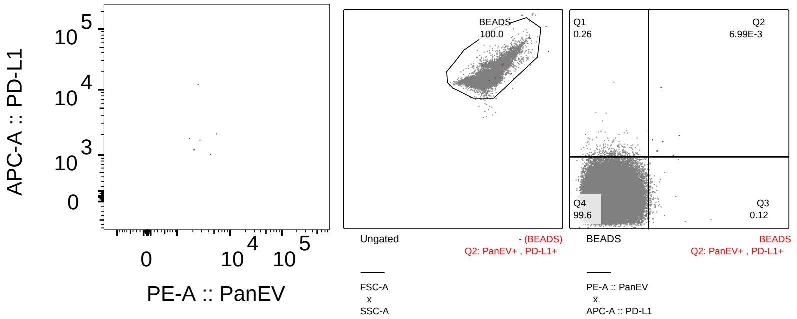

Sample Name	Freq. of BEADS
Specimen_001_049_TP1-5_total_1 ml_IgG_007.fcs	6.99E-3

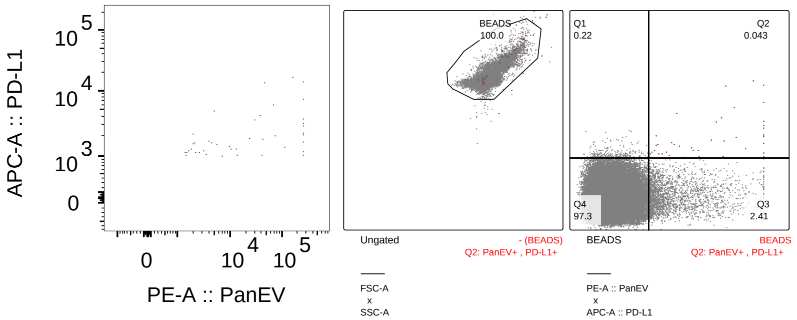

Sample Name	Freq. of BEADS
Specimen_001_049_TP4_1 ml_EV staining_011.fcs	0.043

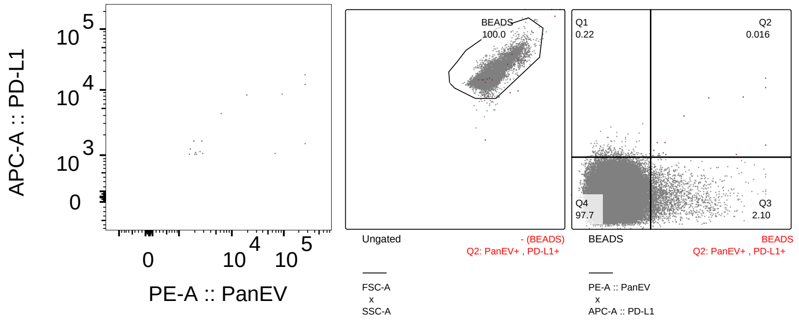

	Sample Name	Freq. of BEADS
▮	Specimen_001_045_TP3_1 ml_EV staining_004.fcs	0.016

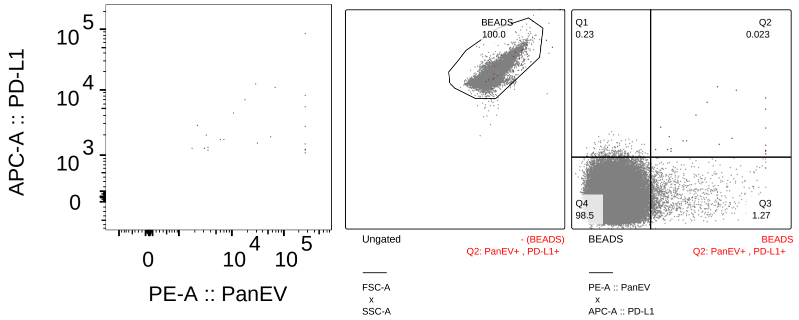

	Sample Name	Freq. of BEADS
▮	Specimen_001_049_TP1_1 ml_EV staining_008.fcs	0.023

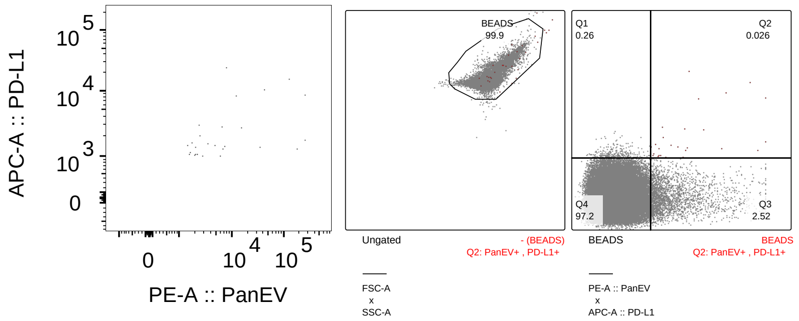

	Sample Name	Freq. of BEADS
▮	Specimen_001_049_TP5_1 ml_EV staining_012.fcs	0.026
