# Supplementary material for: Extracellular vesicles as prognostic biomarkers: results of a neoadjuvant chemoimmunotherapy clinical trial in stage IIIA (N2) non-small-cell lung cancer (SAKK 16/14)
Source: Front Immunol. 2026 Jul 1;17:1807542. doi: 10.3389/fimmu.2026.1807542 (PMC13369264; doi:10.3389/fimmu.2026.1807542)
Supplement: Supplementary Figure 1 — Trial design and extracellular vesicle isolation workflow. Trial design adapted from Rothschild, Sacha I., et al. “SAKK 16/14: durvalumab in addition to neoadjuvant chemotherapy in patients with stage IIIA (N2) non–small-cell lung cancer—a multicenter single-arm phase II trial.” (a) Workflow of extracellular vesicle (EV) isolation and characterization adapted from Benecke, Laura et al. “Isolation and analysis of tumor−derived extracellular vesicles from head and neck squamous cell carcinoma plasma by galectin−based glycan recognition particles.” Created in BioRender. Chiang, M. (2025) https://BioRender.com/7sfvuh0 (b). [file DataSheet1.zip › Gated_Raw_flow_data/(63 + 64) EpCAM+ PanEV+.pdf]

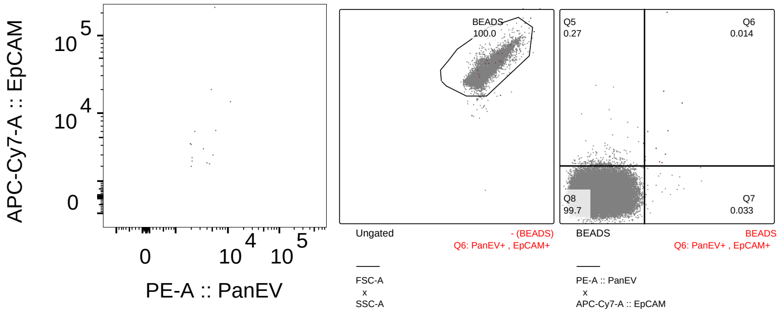

	Sample Name	Freq. of BEADS
	Specimen_001_063_TP1-5_total_1 ml_IgG_001.fcs	0.014

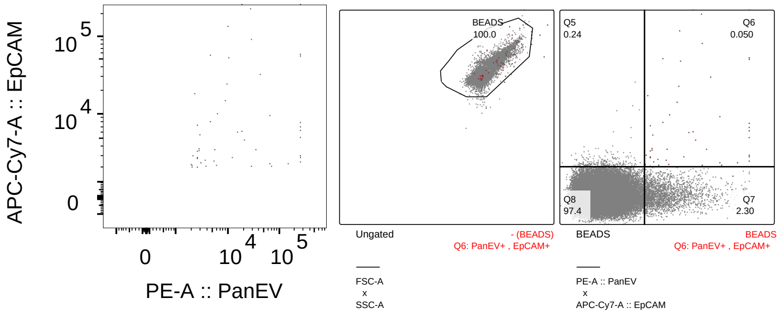

	Sample Name	Freq. of BEADS
	Specimen_001_063_TP4_1 ml_EV staining_005.fcs	0.050

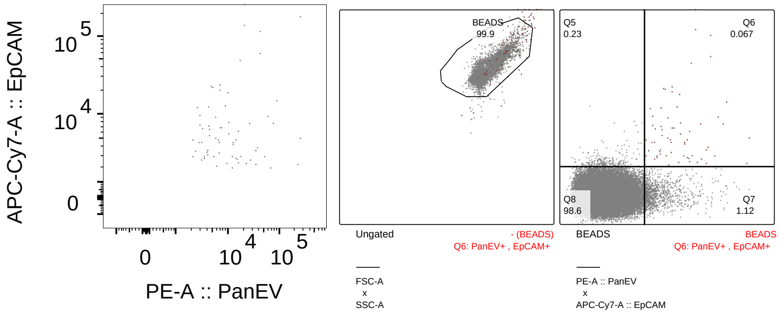

	Sample Name	Freq. of BEADS
	Specimen_001_064_TP2_1 ml_EV staining_009.fcs	0.067

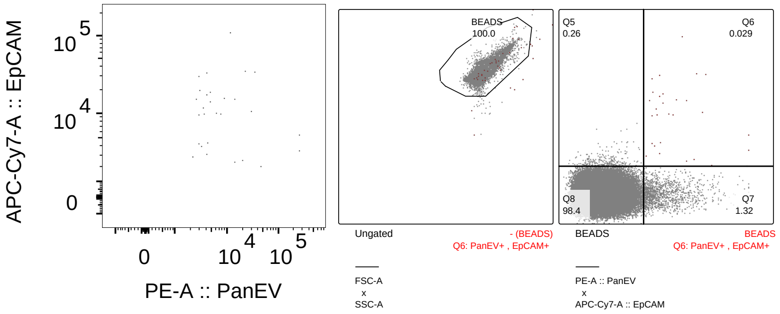

	Sample Name	Freq. of BEADS
	Specimen_001_063_TP1_1 ml_EV staining_002.fcs	0.029

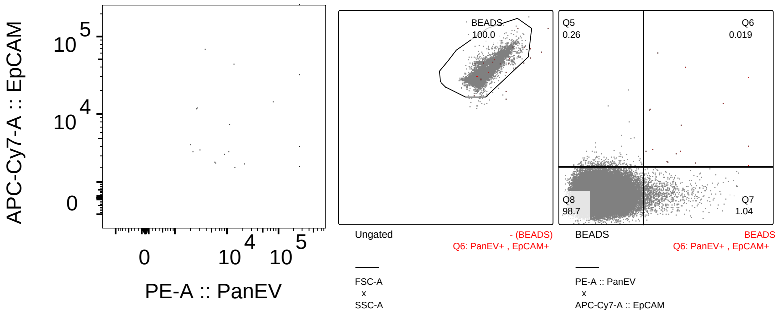

	Sample Name	Freq. of BEADS
	Specimen_001_063_TP5_1 ml_EV staining_006.fcs	0.019

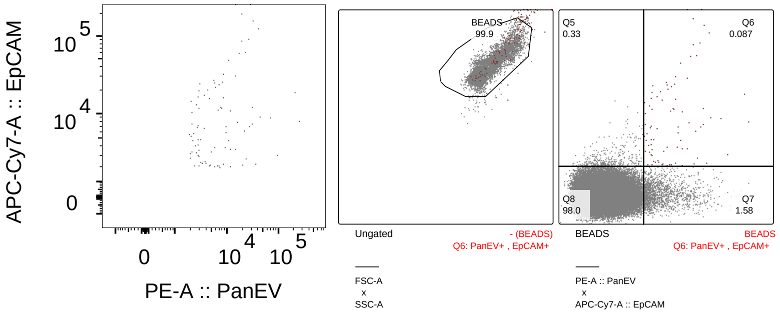

	Sample Name	Freq. of BEADS
	Specimen_001_064_TP3_1 ml_EV staining_010.fcs	0.087

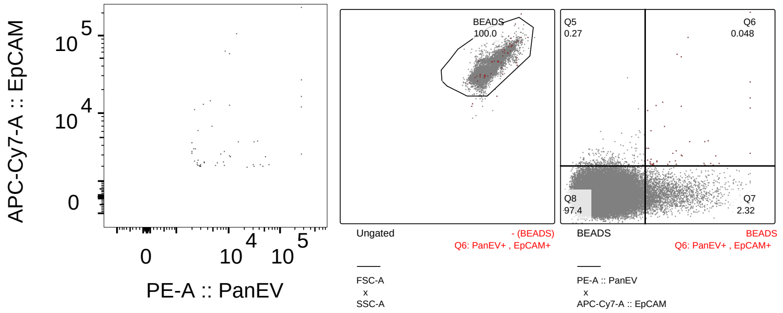

Sample Name	Freq. of BEADS
Specimen_001_063_TP2_1 ml_EV staining_003.fcs	0.048

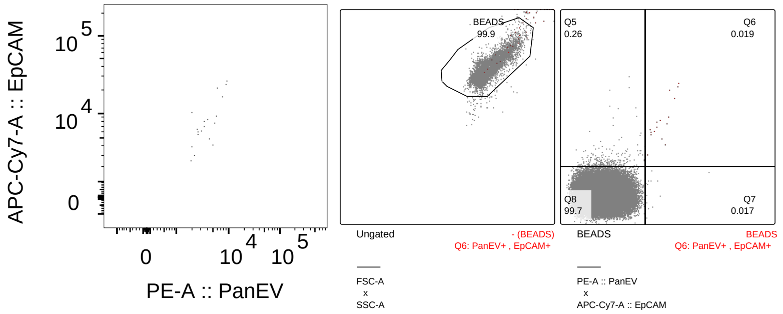

Sample Name	Freq. of BEADS
Specimen_001_064_TP1-5_total_1 ml_IgG_007.fcs	0.019

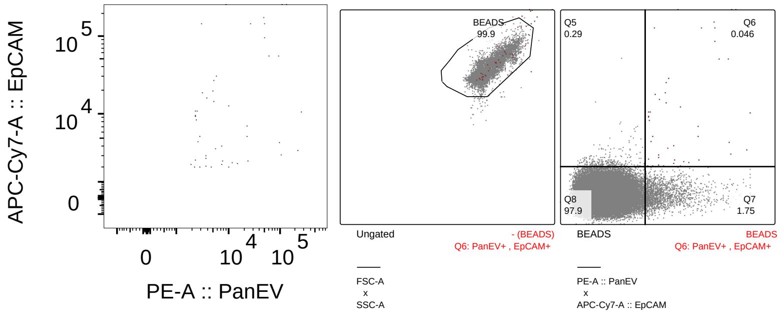

Sample Name	Freq. of BEADS
Specimen_001_064_TP4_1 ml_EV staining_011.fcs	0.046

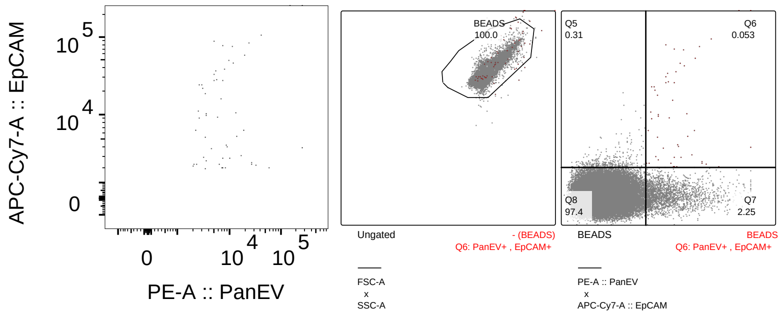

	Sample Name	Freq. of BEADS
	Specimen_001_063_TP3_1 ml_EV staining_004.fcs	0.053

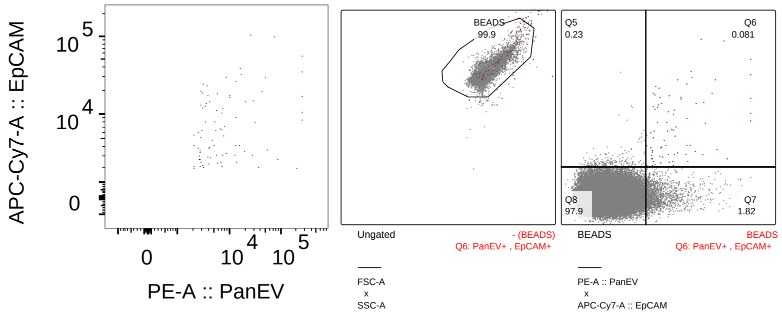

	Sample Name	Freq. of BEADS
	Specimen_001_064_TP1_1 ml_EV staining_008.fcs	0.081

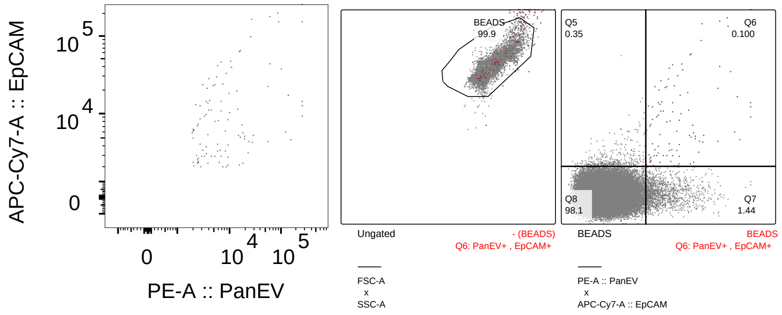

	Sample Name	Freq. of BEADS
	Specimen_001_064_TP5_1 ml_EV staining_012.fcs	0.100
