# Supplementary material for: Extracellular vesicles as prognostic biomarkers: results of a neoadjuvant chemoimmunotherapy clinical trial in stage IIIA (N2) non-small-cell lung cancer (SAKK 16/14)
Source: Front Immunol. 2026 Jul 1;17:1807542. doi: 10.3389/fimmu.2026.1807542 (PMC13369264; doi:10.3389/fimmu.2026.1807542)
Supplement: Supplementary Figure 1 — Trial design and extracellular vesicle isolation workflow. Trial design adapted from Rothschild, Sacha I., et al. “SAKK 16/14: durvalumab in addition to neoadjuvant chemotherapy in patients with stage IIIA (N2) non–small-cell lung cancer—a multicenter single-arm phase II trial.” (a) Workflow of extracellular vesicle (EV) isolation and characterization adapted from Benecke, Laura et al. “Isolation and analysis of tumor−derived extracellular vesicles from head and neck squamous cell carcinoma plasma by galectin−based glycan recognition particles.” Created in BioRender. Chiang, M. (2025) https://BioRender.com/7sfvuh0 (b). [file DataSheet1.zip › Gated_Raw_flow_data/(63 + 64) MFI.pdf]

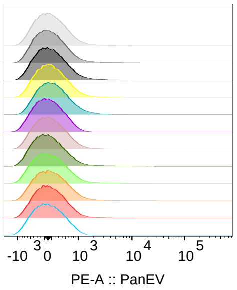

Sample Name	Median : PE-A	Mean : PE-A	Geometric Mean : PE-A
Specimen_001_064_TP5_1 ml_EV staining_012.fcs	58.4	224	99.7
Specimen_001_064_TP4_1 ml_EV staining_011.fcs	44.9	293	90.8
Specimen_001_064_TP3_1 ml_EV staining_010.fcs	58.4	225	102
Specimen_001_064_TP2_1 ml_EV staining_009.fcs	77.9	217	112
Specimen_001_064_TP1_1 ml_EV staining_008.fcs	128	305	169
Specimen_001_064_TP1-5_total_1 ml_IgG_007.fcs	8.97	31.4	24.1
Specimen_001_063_TP5_1 ml_EV staining_006.fcs	41.9	175	74.8
Specimen_001_063_TP4_1 ml_EV staining_005.fcs	13.5	320	70.1
Specimen_001_063_TP3_1 ml_EV staining_004.fcs	37.4	340	88.4
Specimen_001_063_TP2_1 ml_EV staining_003.fcs	76.4	400	126
Specimen_001_063_TP1_1 ml_EV staining_002.fcs	44.9	212	82.5
Specimen_001_063_TP1-5_total_1 ml_IgG_001.fcs	8.97	33.8	25.2

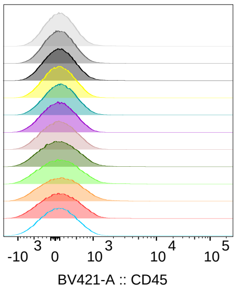

Sample Name	Median : BV421-A	Mean : BV421-A	Geometric Mean : BV421-A
Specimen_001_064_TP5_1 ml_EV staining_012.fcs	113	149	124
Specimen_001_064_TP4_1 ml_EV staining_011.fcs	108	142	115
Specimen_001_064_TP3_1 ml_EV staining_010.fcs	109	143	119
Specimen_001_064_TP2_1 ml_EV staining_009.fcs	103	125	108
Specimen_001_064_TP1_1 ml_EV staining_008.fcs	133	158	137
Specimen_001_064_TP1-5_total_1 ml_IgG_007.fcs	89.9	96.6	88.6
Specimen_001_063_TP5_1 ml_EV staining_006.fcs	97.7	124	102
Specimen_001_063_TP4_1 ml_EV staining_005.fcs	93.8	166	111
Specimen_001_063_TP3_1 ml_EV staining_004.fcs	116	182	129
Specimen_001_063_TP2_1 ml_EV staining_003.fcs	144	219	156
Specimen_001_063_TP1_1 ml_EV staining_002.fcs	112	148	115
Specimen_001_063_TP1-5_total_1 ml_IgG_001.fcs	89.9	95.7	86.6

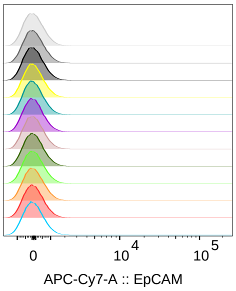

Sample Name	Median : APC-Cy7-A	Mean : APC-Cy7-A	Geometric Mean : APC-Cy7-A
Specimen_001_064_TP5_1 ml_EV staining_012.fcs	-6.64	54.3	32.5
Specimen_001_064_TP4_1 ml_EV staining_011.fcs	-9.96	46.7	27.4
Specimen_001_064_TP3_1 ml_EV staining_010.fcs	-13.3	42.9	24.1
Specimen_001_064_TP2_1 ml_EV staining_009.fcs	-9.96	35.6	24.2
Specimen_001_064_TP1_1 ml_EV staining_008.fcs	-23.3	23.9	15.2
Specimen_001_064_TP1-5_total_1 ml_IgG_007.fcs	-16.6	17.4	13.6
Specimen_001_063_TP5_1 ml_EV staining_006.fcs	-6.64	31.1	24.7
Specimen_001_063_TP4_1 ml_EV staining_005.fcs	-6.64	39.9	25.3
Specimen_001_063_TP3_1 ml_EV staining_004.fcs	-3.32	47.4	29.3
Specimen_001_063_TP2_1 ml_EV staining_003.fcs	-3.32	37.3	29.5
Specimen_001_063_TP1_1 ml_EV staining_002.fcs	-6.64	30.6	25.2
Specimen_001_063_TP1-5_total_1 ml_IgG_001.fcs	-9.96	25.6	20.4

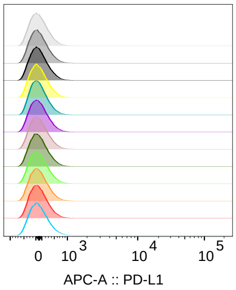

Sample Name	Median : APC-A	Mean : APC-A	Geometric Mean : APC-A
Specimen_001_064_TP5_1 ml_EV staining_012.fcs	-7.15	22.8	18.1
Specimen_001_064_TP4_1 ml_EV staining_011.fcs	-12.5	21.1	15.4
Specimen_001_064_TP3_1 ml_EV staining_010.fcs	-10.7	17.9	15.5
Specimen_001_064_TP2_1 ml_EV staining_009.fcs	-10.7	16.3	14.5
Specimen_001_064_TP1_1 ml_EV staining_008.fcs	-14.3	13.5	11.9
Specimen_001_064_TP1-5_total_1 ml_IgG_007.fcs	-8.94	22.2	17.8
Specimen_001_063_TP5_1 ml_EV staining_006.fcs	-5.36	21.7	19.8
Specimen_001_063_TP4_1 ml_EV staining_005.fcs	-10.7	17.7	15.5
Specimen_001_063_TP3_1 ml_EV staining_004.fcs	-7.15	19.5	17.4
Specimen_001_063_TP2_1 ml_EV staining_003.fcs	-8.94	18.1	16.4
Specimen_001_063_TP1_1 ml_EV staining_002.fcs	-10.7	16.2	14.6
Specimen_001_063_TP1-5_total_1 ml_IgG_001.fcs	-10.7	21.0	16.4

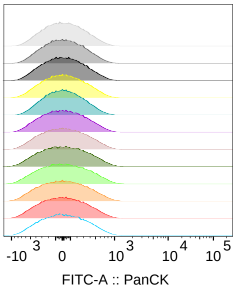

Sample Name	Median : FITC-A	Mean : FITC-A	Geometric Mean : FITC-A
Specimen_001_064_TP5_1 ml_EV staining_012.fcs	16.8	32.0	21.0
Specimen_001_064_TP4_1 ml_EV staining_011.fcs	14.5	28.2	18.7
Specimen_001_064_TP3_1 ml_EV staining_010.fcs	11.5	22.5	16.1
Specimen_001_064_TP2_1 ml_EV staining_009.fcs	15.3	34.5	18.7
Specimen_001_064_TP1_1 ml_EV staining_008.fcs	12.3	22.9	16.6
Specimen_001_064_TP1-5_total_1 ml_IgG_007.fcs	9.95	17.1	13.6
Specimen_001_063_TP5_1 ml_EV staining_006.fcs	12.3	26.5	16.1
Specimen_001_063_TP4_1 ml_EV staining_005.fcs	17.6	38.8	20.7
Specimen_001_063_TP3_1 ml_EV staining_004.fcs	14.5	33.0	18.4
Specimen_001_063_TP2_1 ml_EV staining_003.fcs	9.95	23.7	13.7
Specimen_001_063_TP1_1 ml_EV staining_002.fcs	6.13	18.1	11.7
Specimen_001_063_TP1-5_total_1 ml_IgG_001.fcs	12.3	22.1	17.8
