# Supplementary material for: Extracellular vesicles as prognostic biomarkers: results of a neoadjuvant chemoimmunotherapy clinical trial in stage IIIA (N2) non-small-cell lung cancer (SAKK 16/14)
Source: Front Immunol. 2026 Jul 1;17:1807542. doi: 10.3389/fimmu.2026.1807542 (PMC13369264; doi:10.3389/fimmu.2026.1807542)
Supplement: Supplementary Figure 1 — Trial design and extracellular vesicle isolation workflow. Trial design adapted from Rothschild, Sacha I., et al. “SAKK 16/14: durvalumab in addition to neoadjuvant chemotherapy in patients with stage IIIA (N2) non–small-cell lung cancer—a multicenter single-arm phase II trial.” (a) Workflow of extracellular vesicle (EV) isolation and characterization adapted from Benecke, Laura et al. “Isolation and analysis of tumor−derived extracellular vesicles from head and neck squamous cell carcinoma plasma by galectin−based glycan recognition particles.” Created in BioRender. Chiang, M. (2025) https://BioRender.com/7sfvuh0 (b). [file DataSheet1.zip › Gated_Raw_flow_data/(69 + 73) MFI.pdf]

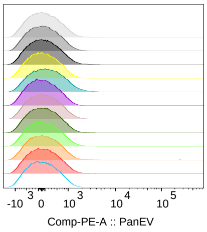

Sample Name	Median : Comp-PE-A	Mean : Comp-PE-A	Geometric Mean : Comp-PE-A
Specimen_001_073 T5 (1 ml 10000g)+ 900 ul PBS_012.fcs	56.9	111	79.3
Specimen_001_073 T4 (1 ml 10000g)+ 900 ul PBS_011.fcs	52.4	133	70.9
Specimen_001_073 T3 (1 ml 10000g)+ 900 ul PBS_010.fcs	58.4	117	80.5
Specimen_001_073 T2 (1 ml 10000g)+ 900 ul PBS_009.fcs	38.9	74.3	55.8
Specimen_001_073 T1 (1 ml 10000g)+ 900 ul PBS_008.fcs	151	235	171
Specimen_001_073 (200ul x5)+ 900 UL PBS (IgG)_007.fcs	22.4	49.8	38.0
Specimen_001_069 T5 (1 ml 10000g)+ 900 ul PBS_006.fcs	65.9	113	83.0
Specimen_001_069 T4 (1 ml 10000g)+ 900 ul PBS_005.fcs	56.9	95.7	71.1
Specimen_001_069 T3 (1 ml 10000g)+ 900 ul PBS_004.fcs	59.9	260	86.3
Specimen_001_069 T2 (1 ml 10000g)+ 900 ul PBS_003.fcs	59.9	579	95.2
Specimen_001_069 T1 (1 ml 10000g)+ 900 ul PBS_002.fcs	56.9	99.3	73.5
Specimen_001_069 (200ul x5)+ 900 UL PBS (IgG)_001.fcs	26.9	58.0	41.2

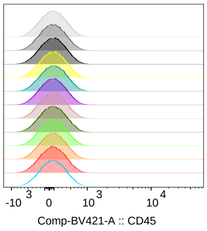

Sample Name	Median : Comp-BV421-A	Mean : Comp-BV421-A	Geometric Mean : Comp-BV421-A
Specimen_001_073 T5 (1 ml 10000g)+ 900 ul PBS_012.fcs	90.7	94.4	89.9
Specimen_001_073 T4 (1 ml 10000g)+ 900 ul PBS_011.fcs	86.3	97.4	86.0
Specimen_001_073 T3 (1 ml 10000g)+ 900 ul PBS_010.fcs	92.9	95.0	90.0
Specimen_001_073 T2 (1 ml 10000g)+ 900 ul PBS_009.fcs	92.9	94.4	89.3
Specimen_001_073 T1 (1 ml 10000g)+ 900 ul PBS_008.fcs	105	110	104
Specimen_001_073 (200ul x5)+ 900 UL PBS (IgG)_007.fcs	88.5	91.2	87.0
Specimen_001_069 T5 (1 ml 10000g)+ 900 ul PBS_006.fcs	95.1	98.6	92.8
Specimen_001_069 T4 (1 ml 10000g)+ 900 ul PBS_005.fcs	91.8	94.9	90.1
Specimen_001_069 T3 (1 ml 10000g)+ 900 ul PBS_004.fcs	96.2	122	96.5
Specimen_001_069 T2 (1 ml 10000g)+ 900 ul PBS_003.fcs	94.0	181	99.0
Specimen_001_069 T1 (1 ml 10000g)+ 900 ul PBS_002.fcs	90.7	97.1	90.0
Specimen_001_069 (200ul x5)+ 900 UL PBS (IgG)_001.fcs	89.6	93.0	88.6

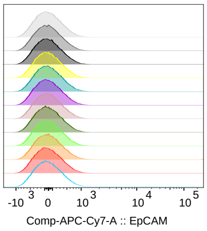

Sample Name	Median : Comp-APC-Cy7-A	Mean : Comp-APC-Cy7-A	Geometric Mean : Comp-APC-Cy7-A
Specimen_001_073 T5 (1 ml 10000g)+ 900 ul PBS_012.fcs	-16.7	2.74	0.59
Specimen_001_073 T4 (1 ml 10000g)+ 900 ul PBS_011.fcs	-12.8	27.9	2.81
Specimen_001_073 T3 (1 ml 10000g)+ 900 ul PBS_010.fcs	-14.1	7.33	3.46
Specimen_001_073 T2 (1 ml 10000g)+ 900 ul PBS_009.fcs	-12.8	7.26	4.60
Specimen_001_073 T1 (1 ml 10000g)+ 900 ul PBS_008.fcs	-10.3	9.33	6.57
Specimen_001_073 (200ul x5)+ 900 UL PBS (IgG)_007.fcs	-15.4	3.80	1.62
Specimen_001_069 T5 (1 ml 10000g)+ 900 ul PBS_006.fcs	-10.3	9.31	5.78
Specimen_001_069 T4 (1 ml 10000g)+ 900 ul PBS_005.fcs	-12.8	6.86	4.32
Specimen_001_069 T3 (1 ml 10000g)+ 900 ul PBS_004.fcs	-8.98	86.2	11.3
Specimen_001_069 T2 (1 ml 10000g)+ 900 ul PBS_003.fcs	-1.28	296	26.0
Specimen_001_069 T1 (1 ml 10000g)+ 900 ul PBS_002.fcs	-16.7	5.47	1.40
Specimen_001_069 (200ul x5)+ 900 UL PBS (IgG)_001.fcs	-18.0	2.30	-0.96

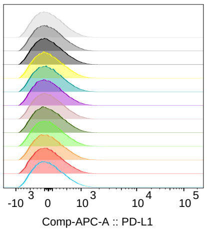

Sample Name	Median : Comp-APC-A	Mean : Comp-APC-A	Geometric Mean : Comp-APC-A
Specimen_001_073 T5 (1 ml 10000g)+ 900 ul PBS_012.fcs	-11.6	26.5	20.0
Specimen_001_073 T4 (1 ml 10000g)+ 900 ul PBS_011.fcs	-7.70	44.4	24.3
Specimen_001_073 T3 (1 ml 10000g)+ 900 ul PBS_010.fcs	-15.4	27.5	20.1
Specimen_001_073 T2 (1 ml 10000g)+ 900 ul PBS_009.fcs	-15.4	26.7	19.9
Specimen_001_073 T1 (1 ml 10000g)+ 900 ul PBS_008.fcs	-11.6	27.7	20.9
Specimen_001_073 (200ul x5)+ 900 UL PBS (IgG)_007.fcs	-14.1	26.0	18.7
Specimen_001_069 T5 (1 ml 10000g)+ 900 ul PBS_006.fcs	-6.42	34.0	26.5
Specimen_001_069 T4 (1 ml 10000g)+ 900 ul PBS_005.fcs	-11.6	30.5	22.0
Specimen_001_069 T3 (1 ml 10000g)+ 900 ul PBS_004.fcs	-7.70	76.7	25.8
Specimen_001_069 T2 (1 ml 10000g)+ 900 ul PBS_003.fcs	-3.85	249	38.2
Specimen_001_069 T1 (1 ml 10000g)+ 900 ul PBS_002.fcs	-14.1	26.8	19.5
Specimen_001_069 (200ul x5)+ 900 UL PBS (IgG)_001.fcs	-14.1	41.3	20.3

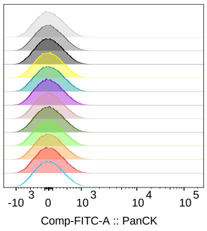

Sample Name	Median : Comp-FITC-A	Mean : Comp-FITC-A	Geometric Mean : Comp-FITC-A
Specimen_001_073 T5 (1 ml 10000g)+ 900 ul PBS_012.fcs	19.3	33.8	30.4
Specimen_001_073 T4 (1 ml 10000g)+ 900 ul PBS_011.fcs	19.3	33.2	29.3
Specimen_001_073 T3 (1 ml 10000g)+ 900 ul PBS_010.fcs	19.3	32.1	28.8
Specimen_001_073 T2 (1 ml 10000g)+ 900 ul PBS_009.fcs	18.0	30.5	27.3
Specimen_001_073 T1 (1 ml 10000g)+ 900 ul PBS_008.fcs	24.4	37.0	33.5
Specimen_001_073 (200ul x5)+ 900 UL PBS (IgG)_007.fcs	15.4	28.8	25.5
Specimen_001_069 T5 (1 ml 10000g)+ 900 ul PBS_006.fcs	20.5	32.3	29.0
Specimen_001_069 T4 (1 ml 10000g)+ 900 ul PBS_005.fcs	19.3	32.3	29.1
Specimen_001_069 T3 (1 ml 10000g)+ 900 ul PBS_004.fcs	19.3	34.8	28.5
Specimen_001_069 T2 (1 ml 10000g)+ 900 ul PBS_003.fcs	24.4	51.6	35.7
Specimen_001_069 T1 (1 ml 10000g)+ 900 ul PBS_002.fcs	21.8	37.2	30.6
Specimen_001_069 (200ul x5)+ 900 UL PBS (IgG)_001.fcs	19.3	31.8	28.3
