# Supplementary material for: Extracellular vesicles as prognostic biomarkers: results of a neoadjuvant chemoimmunotherapy clinical trial in stage IIIA (N2) non-small-cell lung cancer (SAKK 16/14)
Source: Front Immunol. 2026 Jul 1;17:1807542. doi: 10.3389/fimmu.2026.1807542 (PMC13369264; doi:10.3389/fimmu.2026.1807542)
Supplement: Supplementary Figure 1 — Trial design and extracellular vesicle isolation workflow. Trial design adapted from Rothschild, Sacha I., et al. “SAKK 16/14: durvalumab in addition to neoadjuvant chemotherapy in patients with stage IIIA (N2) non–small-cell lung cancer—a multicenter single-arm phase II trial.” (a) Workflow of extracellular vesicle (EV) isolation and characterization adapted from Benecke, Laura et al. “Isolation and analysis of tumor−derived extracellular vesicles from head and neck squamous cell carcinoma plasma by galectin−based glycan recognition particles.” Created in BioRender. Chiang, M. (2025) https://BioRender.com/7sfvuh0 (b). [file DataSheet1.zip › Gated_Raw_flow_data/(69 + 73) PanEV+ CD45-.pdf]

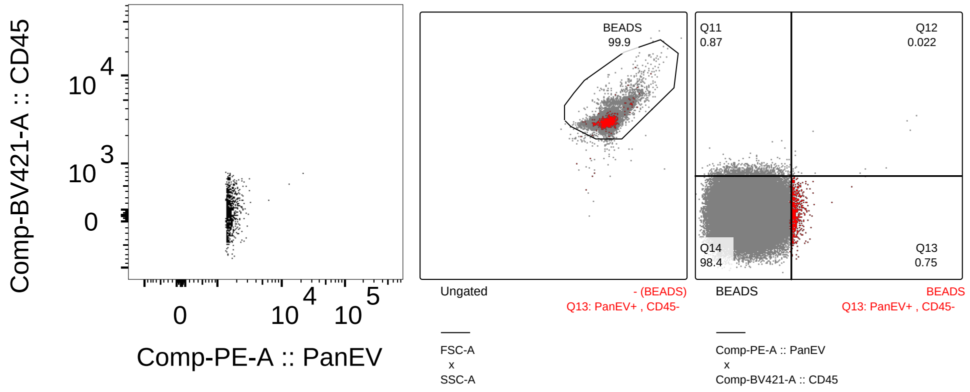

	Sample Name	Freq. of BEADS
■	Specimen_001_069 (200ul x5)+ 900 UL PBS (IgG)_001.fcs	0.75

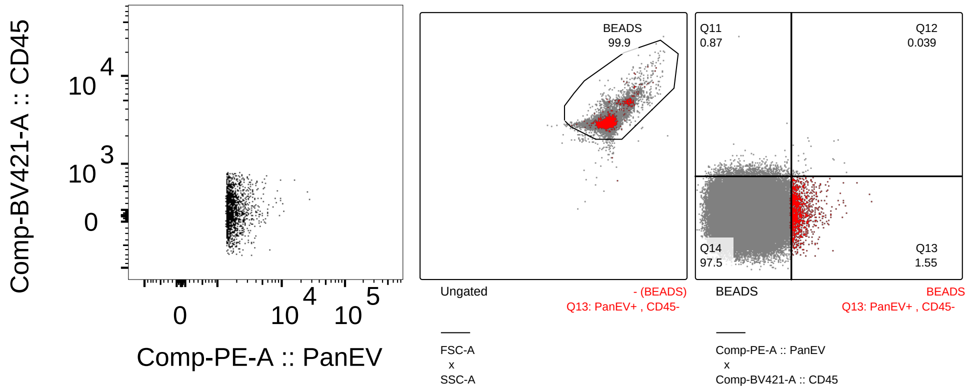

	Sample Name	Freq. of BEADS
■	Specimen_001_069 T4 (1 ml 10000g)+ 900 ul PBS_005.fcs	1.55

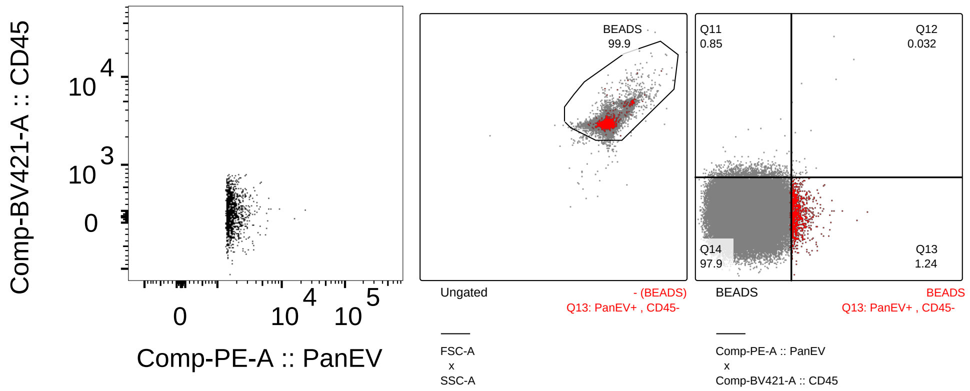

	Sample Name	Freq. of BEADS
■	Specimen_001_073 T2 (1 ml 10000g)+ 900 ul PBS_009.fcs	1.24

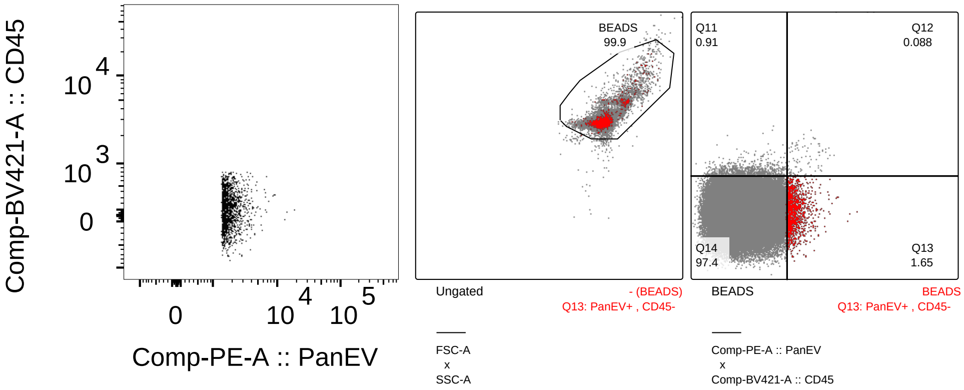

	Sample Name	Freq. of BEADS
■	Specimen_001_069 T1 (1 ml 10000g)+ 900 ul PBS_002.fcs	1.65

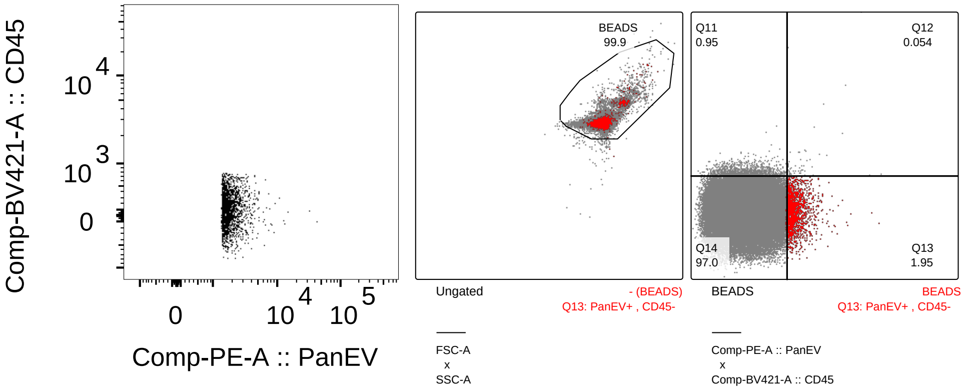

	Sample Name	Freq. of BEADS
■	Specimen_001_069 T5 (1 ml 10000g)+ 900 ul PBS_006.fcs	1.95

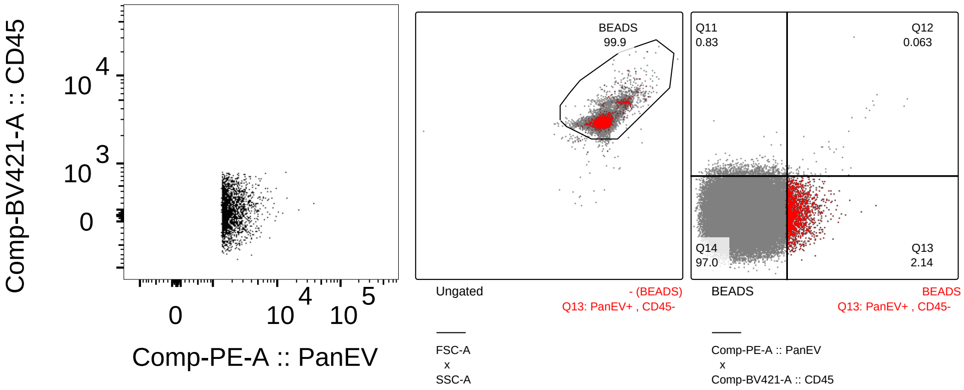

	Sample Name	Freq. of BEADS
■	Specimen_001_073 T3 (1 ml 10000g)+ 900 ul PBS_010.fcs	2.14

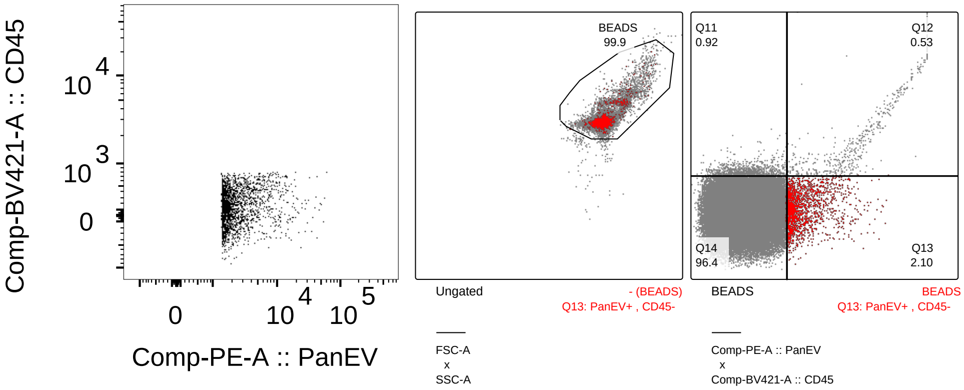

	Sample Name	Freq. of BEADS
	Specimen_001_069 T2 (1 ml 10000g)+ 900 ul PBS_003.fcs	2.10

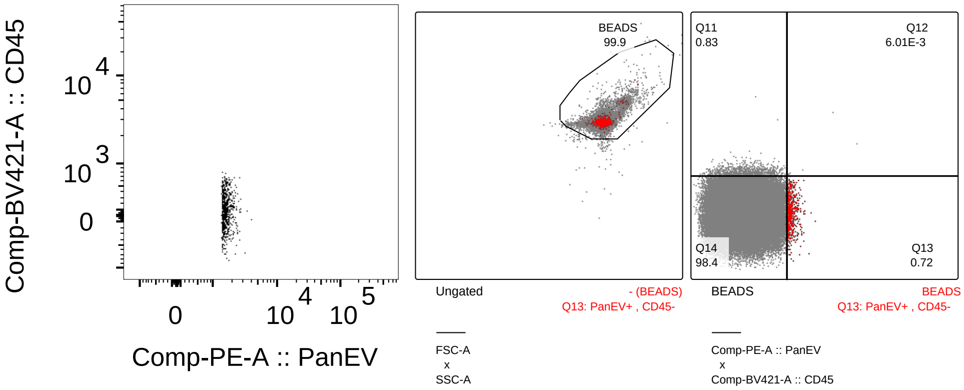

	Sample Name	Freq. of BEADS
	Specimen_001_073 (200ul x5)+ 900 UL PBS (IgG)_007.fcs	0.72

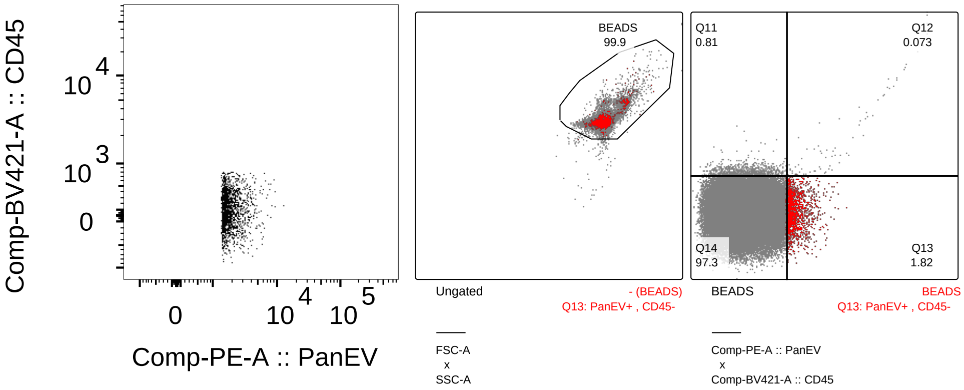

	Sample Name	Freq. of BEADS
	Specimen_001_073 T4 (1 ml 10000g)+ 900 ul PBS_011.fcs	1.82

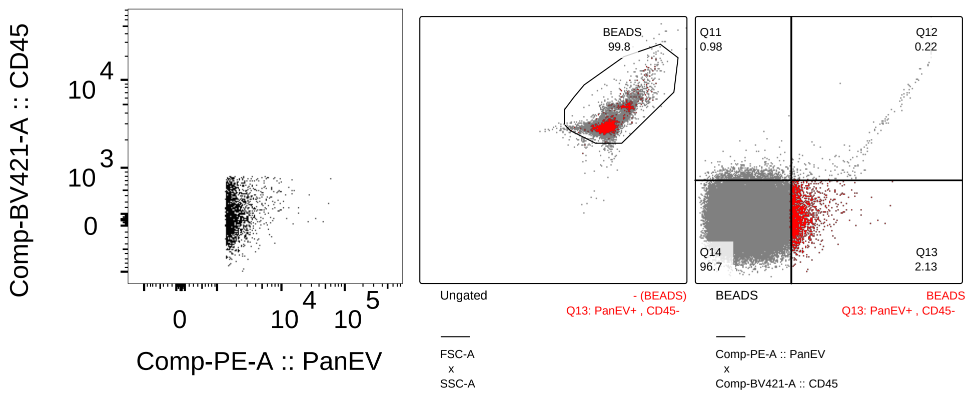

	Sample Name	Freq. of BEADS
■	Specimen_001_069 T3 (1 ml 10000g)+ 900 ul PBS_004.fcs	2.13

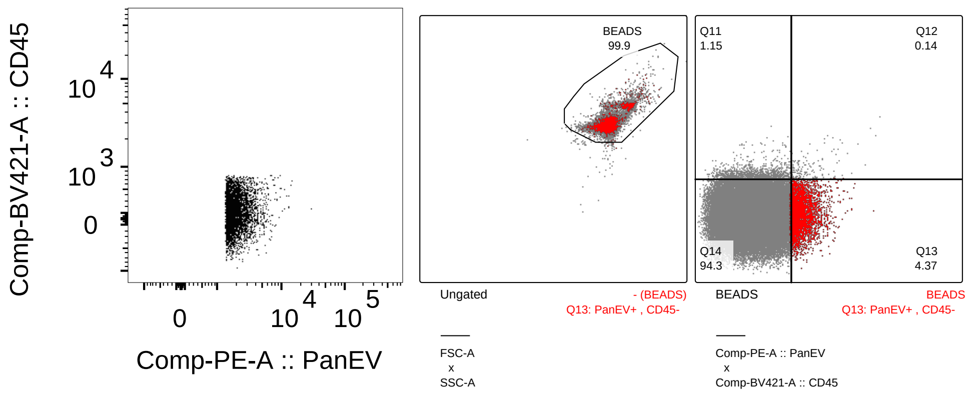

	Sample Name	Freq. of BEADS
■	Specimen_001_073 T1 (1 ml 10000g)+ 900 ul PBS_008.fcs	4.37

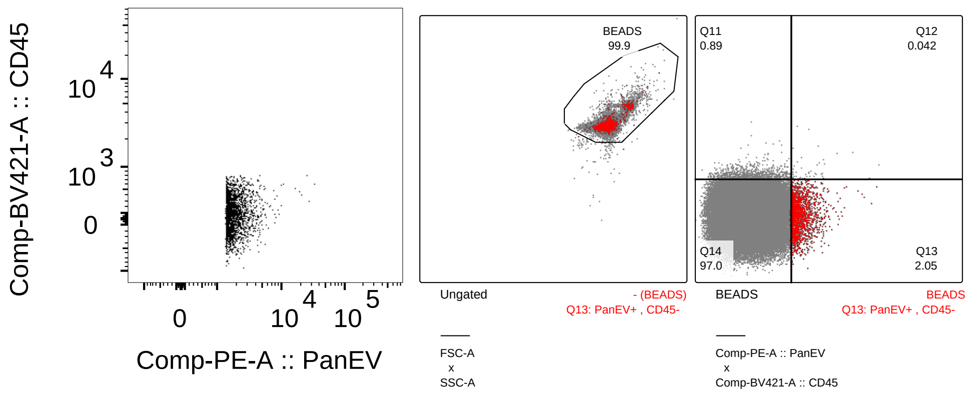

	Sample Name	Freq. of BEADS
■	Specimen_001_073 T5 (1 ml 10000g)+ 900 ul PBS_012.fcs	2.05
