# Supplementary material for: Extracellular vesicles as prognostic biomarkers: results of a neoadjuvant chemoimmunotherapy clinical trial in stage IIIA (N2) non-small-cell lung cancer (SAKK 16/14)
Source: Front Immunol. 2026 Jul 1;17:1807542. doi: 10.3389/fimmu.2026.1807542 (PMC13369264; doi:10.3389/fimmu.2026.1807542)
Supplement: Supplementary Figure 1 — Trial design and extracellular vesicle isolation workflow. Trial design adapted from Rothschild, Sacha I., et al. “SAKK 16/14: durvalumab in addition to neoadjuvant chemotherapy in patients with stage IIIA (N2) non–small-cell lung cancer—a multicenter single-arm phase II trial.” (a) Workflow of extracellular vesicle (EV) isolation and characterization adapted from Benecke, Laura et al. “Isolation and analysis of tumor−derived extracellular vesicles from head and neck squamous cell carcinoma plasma by galectin−based glycan recognition particles.” Created in BioRender. Chiang, M. (2025) https://BioRender.com/7sfvuh0 (b). [file DataSheet1.zip › Gated_Raw_flow_data/(71) EpCAM+ PanEV+.pdf]

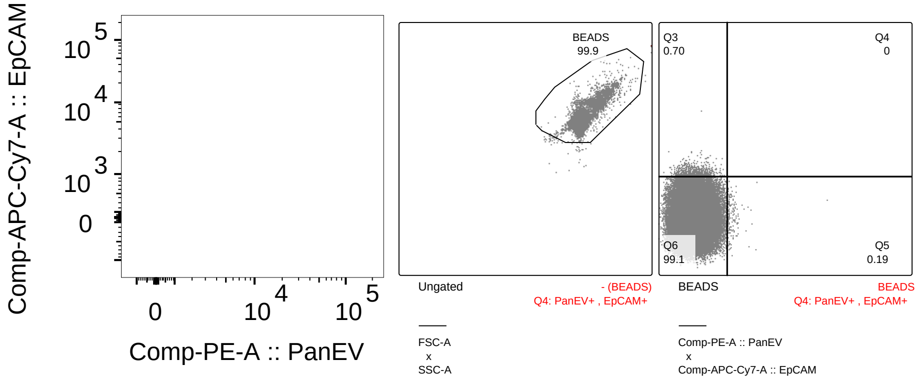

	Sample Name	Freq. of BEADS
█	Specimen_001_071 (200ul x5)+ 900 UL PBS (IgG)_001.fcs	0

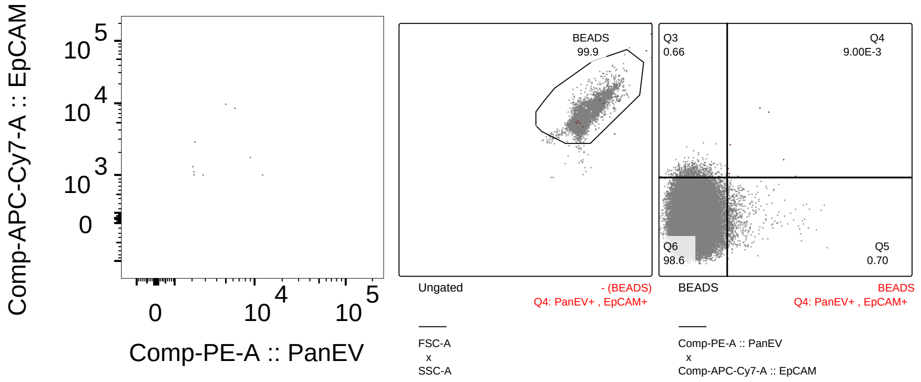

	Sample Name	Freq. of BEADS
█	Specimen_001_071 TP2 1 ml serum+ 900 UL PBS (10000g)_003.fcs	9.00E-3

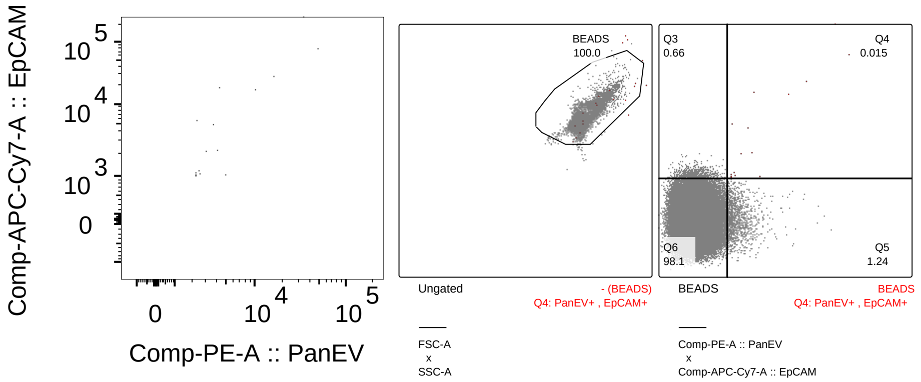

	Sample Name	Freq. of BEADS
█	Specimen_001_071 TP4 1 ml serum+ 900 UL PBS (10000g)_005.fcs	0.015

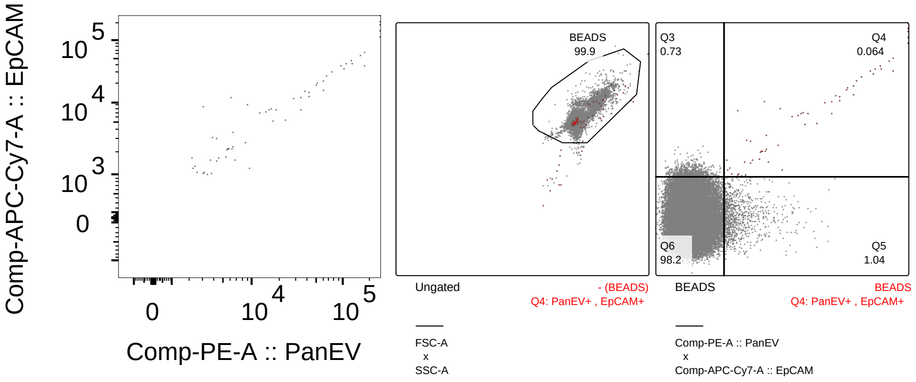

	Sample Name	Freq. of BEADS
	Specimen_001_071 TP1 1 ml serum+ 900 UL PBS (10000g)_002.fcs	0.064

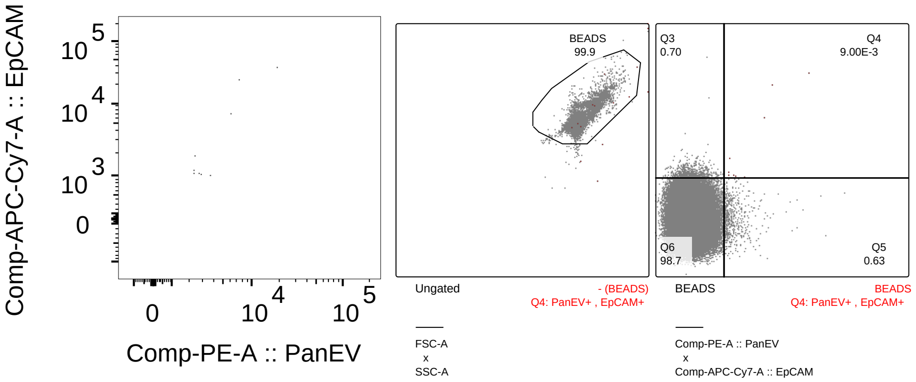

	Sample Name	Freq. of BEADS
	Specimen_001_071 TP3 1 ml serum+ 900 UL PBS (10000g)_004.fcs	9.00E-3

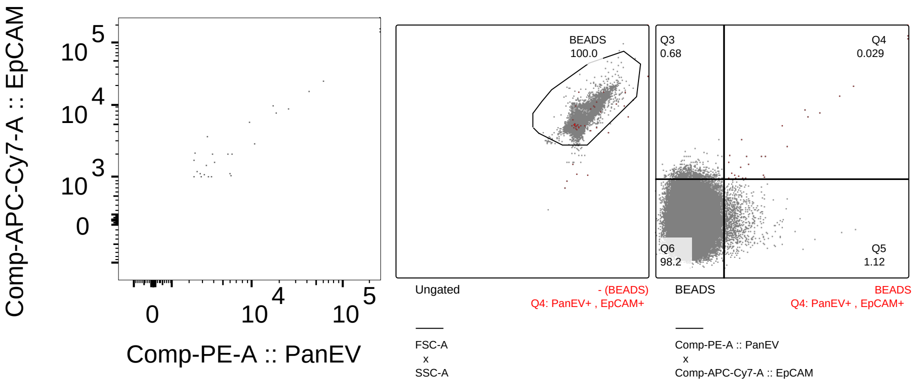

	Sample Name	Freq. of BEADS
	Specimen_001_071 TP5 1 ml serum+ 900 UL PBS (10000g)_006.fcs	0.029
